# Insights of the *Neofusicoccum parvum*–*Liquidambar styraciflua* Interaction and Identification of New Cysteine-Rich Proteins in Both Species

**DOI:** 10.3390/jof7121027

**Published:** 2021-11-30

**Authors:** Rebeca Vázquez-Avendaño, José Benjamín Rodríguez-Haas, Hugo Velázquez-Delgado, Greta Hanako Rosas-Saito, Eric Edmundo Hernández-Domínguez, Diana Sánchez-Rangel

**Affiliations:** 1 Laboratorios de Biología Molecular y Fitopatología, Red de Estudios Moleculares Avanzados, Instituto de Ecología A.C., Xalapa 91070, Veracruz, Mexico; rebe_vazaven@hotmail.com (R.V.-A.); benjamin.rodriguez@inecol.mx (J.B.R.-H.); hugovelazquezdelgado@outlook.es (H.V.-D.); 2 Laboratorio de Microscopía Avanzada, Red de Estudios Moleculares Avanzados, Instituto de Ecología A.C., Xalapa 91070, Veracruz, Mexico; greta.rosas@inecol.mx; 3 Laboratorio de Proteómica, Red de Estudios Moleculares Avanzados, Instituto de Ecología A.C., Xalapa 91070, Veracruz, Mexico; 4 Catedrático CONACyT en la Red de Estudios Moleculares Avanzados, Instituto de Ecología A.C., Xalapa 91070, Veracruz, Mexico

**Keywords:** *Neofusicoccum parvum*, *Liquidambar styraciflua*, cysteine-rich proteins, CFEM domain-containing protein, plant fungal infection

## Abstract

*Neofusicoccum parvum* belongs to the *Botryosphaeriaceae* family, which contains endophytes and pathogens of woody plants. In this study, we isolated 11 strains from diseased tissue of *Liquidambar styraciflua*. Testing with Koch’s postulates—followed by a molecular approach—revealed that *N. parvum* was the most pathogenic strain. We established an in vitro pathosystem (*L. styraciflua* foliar tissue–*N. parvum*) in order to characterize the infection process during the first 16 days. New CysRPs were identified for both organisms using public transcriptomic and genomic databases, while mRNA expression of CysRPs was analyzed by RT-qPCR. The results showed that *N. parvum* caused disease symptoms after 24 h that intensified over time. Through in silico analysis, 5 CysRPs were identified for each organism, revealing that all of the proteins are potentially secreted and novel, including two of *N. parvum* proteins containing the CFEM domain. Interestingly, the levels of the CysRPs mRNAs change during the interaction. This study reports *N. parvum* as a pathogen of *L. styraciflua* for the first time and highlights the potential involvement of CysRPs in both organisms during this interaction.

## 1. Introduction

A current challenge in the area of plant–pathogen interactions is to comprehensively understand the molecular mechanisms involved in the defense response of plants against a specific pathogen. Improved prevention and diagnostic strategies should therefore be designed for more effective disease management. Fungi are important causal agents of plant diseases, and different species among the *Botryosphaeriaceae* family are recognized as endophytic and latent pathogens of trees and other woody plants, and are responsible for causing cankers, dieback, and even blight [1]. *Neofusicoccum parvum* belongs to the *Botryosphaeria* genus and has been recognized as a widespread pathogen worldwide [2], causing damage to a wide range of hosts, including agricultural, horticultural, and forestry plants. Its hosts of economic importance include grapevine (*Vitis vinifera*) [3], avocado (*Persea americana* Mill.) [4,5,6], blueberry (*Vaccinium spp*.) [7], pomegranate (*Punica granatum*) [8], peach (*Prunus persica*) [9], and walnut (*Juglans regia*) [10]. Its forestry host plants include the coast redwood (*Sequoia sempervirens*) [11], Norfolk Island pine (*Araucaria heterophylla*) [12], *Eucalyptus globulus* [13,14], and *Ginkgo biloba* [15]. Most of the relevant studies published to date have focused on isolation of the fungus from symptomatic or asymptomatic tissue, followed by molecular identification and pathogenicity testing using Koch’s postulates. Genomic analysis of UCR-NP2 isolated from grapevine in which the presence of glycoside hydrolases, polysaccharide lyases, cutinases, lignin peroxidases, and cytochrome P450 monooxygenases that might be involved in lignin degradation, and likely also in pathogenesis, were revealed [16]. It is already known that *N. parvum* is able to produce a variety of phytotoxic compounds that belong to the chemical families of dihydrotoluquinone, epoxy lactone, dihydroisocoumarin, and hydroxybenzoic acid, such as (-)-terremutin, (R)-mellein, and 6-methyl-salicylic acid [17]. Recently, exogenous application of (R)-mellein and (-)-terremutin in *V. vinifera* produced upregulation of the salicylic acid (SA)-responsive genes PR1 and GST1. In contrast, the expression of jasmonic acid (JA)/ethylene (ET)-responsive genes remained weak or was even downregulated by (-)- terremutin [18]. Necrosis and ethylene-inducing proteins (NLPs) have recently been identified in this fungus and, using biotechnological approaches, pure recombinant proteins were determined that are toxic to both plant and mammalian cells [19]. To date, there has been no effective control strategy against *N. parvum*, and horizontal transmission of this pathogen [20] has been considered to increase the risk of infection in different hosts. This study reports for the first time that *N. parvum* is a pathogen of *L. styraciflua* and, through molecular approaches, provides insights into this interaction. *Liquidambar styraciflua* L. (*Altingiaceae*) is a deciduous timber tree native to the Americas that is distributed in localized regions in North and Central America [21], and has been widely introduced in eastern and central China [22]. This tree, commonly known as American sweetgum, is an attractive hardwood species for potential bioenergy production [23] and is often used for reforestation, agroforestry, and landscaping [24]. 

Study of specific proteins in both host and pathogen is a good approach to gain knowledge of the interaction. Small proteins with a high percentage of cysteine residues that possess novel functions during plant pathogen interaction are an attractive group of proteins [25]. Examples of these kind of cysteine-rich proteins (CysRPs) include SCR96 of *Phytophthora cactorum* that trigger cell death in *Nicotiana benthamiana* and tomato, silenced transformants of scr96 lose their pathogenicity on host plants [26]. The cysteine-rich small protein SsSSVP1 in *Sclerotinia sclerotiorum* induce plant cell death when interacting with QCR8, a subunit of cytochrome b-c1 complex, and disturbs the localization of QCR8 in mitochondria, suggesting that SsSSVP1 manipulate plant energy metabolism to facilitate the infection of *S. sclerotiorum* [27]. For *Verticillium dahliae*, three CysRPs: VdSCP27, VdSCP113, and VdSCP126 expressed in *Nicotiana benthamiana* caused cell death, a reactive oxygen species burst, and the induction of defense genes [28].

In turn, during the defense response, the plants can produce proteins rich in cysteines with antifungal activity, such as defensins, lipid-transfer proteins, snakins, hevein-like peptides, knottins, hairpinins [29], and cyclopeptides [30]. Snakins/GASA proteins StSN1 from potato and MsSN1 from alfalfa, showed antifungal activity against many fungi and overexpression of StSN1 in potato and wheat improved plant resistance to commercially important pathogens [31]. Hevein-like peptides of *Ginkgo biloba* inhibited the hyphal growth of *Aspergillus niger*, *Curvularia lunata*, *Fusarium oxysporum*, and *Rhizoctonia solani* [32], and finally cyclotides cyO2, cyO3, cyO13, cyO19 of *Viola odorata* showed a potent activity against *Fusarium oxysporum*, *Fusarium graminearum*, *Fusarium culmorum*, *Mycosphaerella fragariae,* and *Botrytis cinerea* [30].

Here, we established a reproducible in vitro pathosystem using foliar tissue of *L. styraciflua* and characterized the infection process using scanning electron microscopy (SEM). We also identified cysteine-rich proteins (CysRPs) of both the plant and the fungus that may be important to the defense response and infection process. Evidence indicates that effector proteins are highly specific and inherent to each pathogen. Finally, no information has been available regarding the CysRPs in any of the species of the *Botryosphaeriaceae* family. 

## 2. Materials and Methods 

### 2.1. Plant Material

*Liquidambar styraciflua* leaves with disease symptoms such as chlorosis and necrosis were sampled from five different areas with trees located within the cloud forest of Santuario del Bosque de Niebla (SBN) in Xalapa, Veracruz, Mexico (19°30′37″ N 96°56′34″ W). This forest is a protected area belonging to the Institute of Ecology (INECOL A.C.) and is used to collect samples for research purposes. Identification of *Liquidambar* specimens was performed by the curators Carlos Duran and Sergio Avendaño in the Herbarium-XAL of INECOL A.C. Leaves and stems used for artificial inoculation were collected from healthy trees in SBN.

### 2.2. Isolation of Fungi from L. styraciflua Leaves

Complete leaves were cut with gardening scissors, deposited in plastic bags, and kept at 4 °C. The leaves were cut into small pieces of 0.5 cm × 0.5 cm and disinfected with ethanol (96%) and chlorine solution (20%) for 2 min each rinsed five times with sterile water. For fungal isolation, the disinfected tissue was placed in Petri dishes containing potato dextrose agar (PDA) and incubated for 2–4 days at 25 or 29 °C under two different light conditions: darkness or a 12 h light/12 h dark. Monosporic cultures were grown for all of the isolated fungi that showed different morphologies. The isolates were preserved in 10% glycerol at −80 °C.

### 2.3. DNA Extraction and Molecular Identification of the Liqui 1–3–Isolate by PCR

Each isolate was grown on PDA in Petri dishes of diameter 10 cm for 9–10 days at 28 °C in darkness. The mycelium was collected with a scalpel and pulverized to isolate genomic DNA according to the protocol described by Tapia-Tussell et al. [33], with minor modifications. Specifically, in the lysis step, we added 1 mL of SDS buffer, while the purification step was replaced with the addition of 500 µL of phenol:chloroform:isoamyl alcohol (Sigma-Aldrich, St. Louis, MO, USA). For molecular identification of the Liqui 1−3 isolate, previously published primers that amplify regions of the β-tubulin gene [34]; the internal transcribed spacer (ITS) regions; a portion of the RNA polymerase II subunit (RPB2) [35]; the BotF15 locus, an unknown locus containing microsatellite repeats [36]; and the portion of the gene encoding translation elongation factor 1 alpha (EF-1α) [35] (see Table S1) were used. Genes from these five regions were amplified from genomic DNA using PCR with Platinum^®^ Taq DNA Polymerase, High Fidelity (Invitrogen™ Thermo Fisher Scientific, Waltham, MA, USA), following the manufacturer’s recommendations. The optimal PCR conditions on a SureCycler 8800 (Agilent Technologies, Inc., Santa Clara, CA, USA) were as follows: initial denaturation at 94 °C for 1 min, followed by 32 cycles of 94 °C for 15 s, 58 °C (β-tubulin, RPB2 and Botf15)/55 °C (ITS and EF-1α) for 30 s, and 72 °C for 1 min, with a final extension step of 72 °C for 5 min. The size and concentration of the amplicons were visually determined by electrophoresis on a 1% agarose gel, and PCR products were purified using the Wizard SV Gel and PCR Clean-up System (Promega, Madison, WI, USA). The DNA concentration was measured using a NanoDrop 2000c spectrophotometer (Thermo Fisher Scientific, USA). Samples were sent to Langebio, Cinvestav, for sequencing. DNA sequences were analyzed using the nucleotide collection (nr/nt), optimized for highly similar sequences (MegaBLAST) in NCBI (https://blast.ncbi.nlm.nih.gov/Blast.cgi, 26 June 2019).

### 2.4. Pathogenicity Assay in Arabidopsis Thaliana Seedlings

Seeds of *A. thaliana* ecotype Col-0 were surface-disinfected with 96% (*v/v*) ethanol for 7 min and 20% (*v/v*) bleach for 7 min, rinsed five times for 5 min with distilled water and stratified for 2 days at 4 °C. Seeds were grown on each agar plate (10 seeds per plate) containing 0.2 × Murashige and Skoog medium (MS basal salt mixture, PhytoTechnology Laboratories, Lenexa, KS, USA), 0.6% sucrose (Merck, Darmstadt, Germany) and 1% Agar Plant TC at pH 7. Plates were incubated at 21 ± 1 °C under a cycle of 12 h light/12 h dark for 7 days. The seedlings were then inoculated with 0.5 cm × 0.5 cm agar plugs containing the isolate, and the plates were incubated for seven additional days under the same conditions. A photograph was then taken with a camera D3200 (Nikon, Tokyo, Japan). All of the experiments were carried out in triplicate.

### 2.5. Pathogenicity Assay in L. styraciflua Leaves and Stems

Healthy *L. styraciflua* leaves and stems were collected from the cloud forest of the SBN at Xalapa, Veracruz, Mexico. Once collected, the samples were immediately placed in plastic bags that contained sterile water to preserve the moisture. The leaves were collected and sterilized on the same day that the experiment was conducted. The leaves were sterilized with 2% sodium hypochlorite for 1 min and then washed five times with sterile distilled water. Three sterilized leaves were placed in a humid chamber. The humid chambers were prepared by placing a circle of sterile filter paper in the bottom of a Petri dish (150 × 20 mm) that had been previously sterilized with UV light for 15 min. Approximately 4–5 mL of sterile distilled water was added to the filter paper. The leaves (3) were mechanically damaged at the base with a sterile scalpel and then inoculated with a plug (approximately 0.5 × 0.5 cm) of *N. parvum* (Liqui 1–3) previously cultured on PDA medium. Leaves with damaged base were used as controls. Finally, the control and inoculated leaves were incubated at 25 ± 2 °C with 80% relative humidity in darkness in a plant growth chamber (Thermo Scientific, USA). Photographs were taken at 0, 1, 3, 8, and 18 days post infection (dpi) with a camera D3200 (Nikon, Japan). Stem infection experiments were carried out as follows: previously sterilized young branches of an adult *L. styraciflua* tree were cut with a scalpel, or single-edged knife, into fragments of approximately 10 cm, and then cut longitudinally. The *L. styraciflua* stems were placed facing upward in quadruplicate inside a humid chamber prepared as previously described. The plant stems were inoculated with 0.5 × 0.5 cm mycelium plugs of *N. parvum* (LSH1-083) previously cultured on PDA medium, at the center of the vascular tissue. A PDA plug with no fungus was included as a negative control. Finally, the sample was incubated at 25 ± 2 °C at 80% relative humidity in darkness in a plant growth chamber (Thermo Scientific, USA).

### 2.6. DAB Staining

Infected and uninfected *L. styraciflua* leaves were stained following a previously reported protocol [37].

### 2.7. SEM

Sections of infected and uninfected *L. styraciflua* leaves, at 1, 3, and 8 dpi, were fixed in 2.5% glutaraldehyde for 12 h and then washed in 0.1 M Sorensen’s phosphate buffer. The samples were dehydrated in an ethanol solution series from 30 to 100% for 40 min each, dried in a critical point drier K850 (Quorum Technologies Ltd., Laughton, UK), coated with gold in a rotary pumped sputter coater Q150RS (Quorum Technologies Ltd., UK) [38], and then examined under a scanning electron microscope Quanta^TM^ FEG 250 (Thermo Fisher Scientific, USA).

### 2.8. CysRP Identification

For identification of the CysRPs of *L. styraciflua* and *N. parvum*, the Hardwood Genomics Project database (https://www.hardwoodgenomics.org/; accession: PRJNA273273) and the genomic information already published by Blanco et al. [16] (accession: PRJNA244484) were consulted, respectively. Prediction of subcellular localization for all proteins, as well as signal peptide cleavage, were performed with the TargetP 1.1 server [39] and Protter [40]. The predicted localization was evaluated with SignalP-5.0. [41] and DeepLoc-1.0 [42]. BLAST (National Center for Biotechnology Information; https://blast.ncbi.nlm.nih.gov/Blast.cgi), Motif search (GenomeNet; https://www.genome.jp/tools/motif/MOTIF.html) and DeepGOPlus [43] approaches were used to infer the possible functions of the CysRPs. Theoretical molecular weight and isoelectric point were predicted with compute pI/Mw tool (https://web.expasy.org/compute_pi) [44].

### 2.9. Phylogenetic Trees

Phylogenetic analysis was performed with Mega X software [45] using the maximum likelihood method and JTT matrix-based model [46]. Jalview software was used to perform the alignments [47] with the T-Coffee method [48] and, finally, the BLAST tool was used to find similar sequences in other fungi.

### 2.10. RNA Extraction and Gene Expression

Total RNA of whole leaves at 24 and 72 h infected and no infected (but mechanically injury) was extracted using the Plant/Fungi Total RNA Purification Kit (Norgen BioTek Corp., Thorold, ON, Canada), following the manufacturer’s recommendations. The concentration of RNA was determined with a NanoDrop™ 2000c spectrophotometer (Thermo Fisher Scientific, USA), and RNA integrity was evaluated based on the A260 nm/A280 nm ratio and by electrophoresis in a 1.5% agarose gel. One µg of the RNA was then treated with deoxyribonuclease I from Invitrogen™ (Thermo Fisher Scientific, USA), following the manufacturer’s instructions, after which the RNA was used as a template for synthesis of cDNA with the reverse transcriptase Superscript III Invitrogen™, again following the manufacturer’s protocols.

Quantitative real-time PCR (qRT-PCR) was carried out using SYBR™ Green PCR Master Mix (Thermo Fisher Scientific, USA) in a final reaction volume of 20 μL, containing 10 μL of SYBR™ Green (Thermo Fisher Scientific, USA), 1.0 μL of each primer (10 mM), and 8 μL of cDNA (50 ng or 5.0 ng). For determination of oligo efficiency, curves with 50.0, 5.0, 0.5, 0.05, and 0.005 ng of cDNA were generated. Real-time PCR was performed in a Stratagene Mx300P system (Agilent Technologies, USA) under the standard thermal profile design: 10 min at 95 °C, followed by a total of 40 cycles of 30 s at 95 °C, 1 min at 55 °C and 1 min at 72 °C. Oligo specificity was determined by a melting curve analysis with continuous fluorescence data acquisition during the 55–95 °C melt. The software Primer3 was used for oligo design, following the parameters suggested by Thornton and Basu [49]. Relative expression levels for validated genes were calculated by the equation [50];

$$ratio=\frac{{\left(E_{target}\right)}^{\Delta CP_{target}\left(control-Sample\right)}}{{\left(E_{ref}\right)}^{\Delta CP_{ref}\left(control-Sample\right)}}$$

and oligos for Tip41, actin and ubiquitin of *L. styraciflua* were evaluated as reference genes (all primers are listed in Table S1). The geometric mean [51] between Tip41 and actin was used as a references [52,53]. 

## 3. Results

### 3.1. Identification of N. parvum as a Pathogen of L. styraciflua

**Figure 1:**
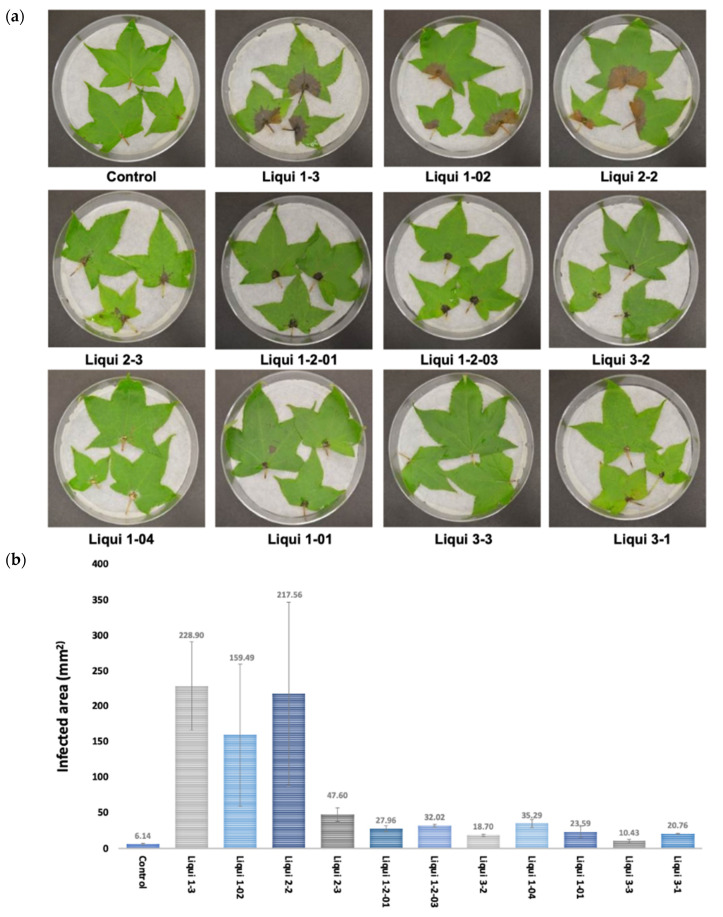
Pathogenicity screening of the different fungal isolates in *L. styraciflua* leaves. (**a**) *Liquidambar styraciflua* leaves were collected from adult trees and infected with different fungal isolates. The photograph was taken at 7 dpi. The data are representative of three independent experiments. (**b**) The graph presents the area values of damaged tissue in mm^2^.

Eleven fungi were isolated from *L. styraciflua* leaves with visible symptoms, such as necrosis and discoloration. The potential pathogenicity of the fungi was tested in *L. styraciflua* leaves and in seedlings of the model plant, *A. thaliana* (Figure 1 and Figure S1). In *L. styraciflua*, Liqui 1–3 provoked clear necrosis and leaf discoloration, including zones at the main veins and petiole, at 8 dpi. Liqui 1–02 and Liqui 2–2 also triggered disease symptoms in *L. styraciflua*; however, in contrast to the effect produced by Liqui 1–3, no noticeable change was observed in petiole coloration. The calculated percentage of damage (relationship between the size of the leaf and the size of the lesion) showed no statistically significant differences among the three strains: a value of 26.8 ± 4.7% damaged leaf area was found for Liqui 1–3, while the values were 15.1 ± 8.5% and 20.1 ± 1.2% for Liqui 1–02 and Liqui 2–2, respectively.

In *A. thaliana* seedlings, Liqui 1–3 covered all of the plant tissue, inducing severe leaf discoloration at 7 dpi. Interestingly, in *A. thaliana* seedlings, Liqui 1–02 and Liqui 2–2 did not have a critical pathogenic effect. For example, while Liqui 2–2 covered more than 50% of the seedlings, the foliar tissue of the infected plants showed greater vigor than that of the control. Liqui 1–2–01, Liqui 1–2–03, and Liqui 3–2 at 7 dpi showed a different pathogenic effect: *Arabidopsis thaliana* plants developed shorter primary roots but an increased number of secondary roots during the interaction with these isolates were observed. The isolates Liqui 1–04, Liqui 1–01, Liqui 3–3, and Liqui 3–1 had no effect on *L. styraciflua* leaves or *A. thaliana* seedlings. Since Liqui 1–3 was the only strain that showed a clear pathogenic effect on both *L. styraciflua* and *A. thaliana* plants, we identified this strain at molecular level and established the pathosystem in *L. styraciflua* leaves. To identify the Liqui 1–3 strain at the molecular level, universal and specific primers were used (Figure S2 and Table S1). The analysis revealed that Liqui 1–3 belongs to the *Botryosphaeriaceae* family as a species *N. parvum*.

### 3.2. Establishment of the L. styraciflua–N. parvum Pathosystem

**Figure 2:**
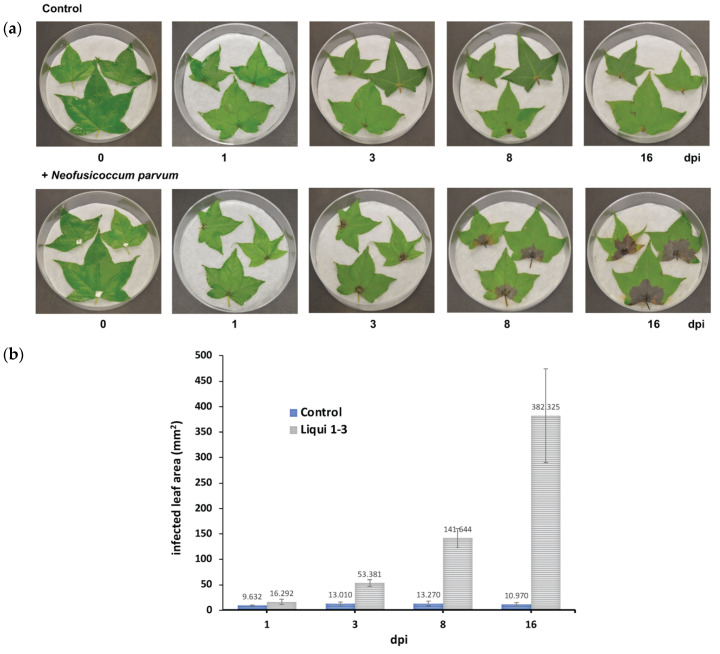
The *N. parvum* and *L. styraciflua* pathosystem. (**a**) The photographs were taken at 0, 1, 3, 8, and 16 dpi. Data are representative of at least three independent experiments. (**b**) The graph presents the leaf damage measured (mm^2^) at 3, 8, and 16 dpi.

Since we were interested in studying the interaction between *L. styraciflua* and *N. parvum*, we established a pathosystem using the *L. styraciflua* leaves. The graph representing the infected leaf area per dpi shows that the infection process progresses rapidly. The inoculated leaves displayed a brownish discoloration at 1 and 3 dpi, which was accentuated over time (Figure 2), and there was visible distinct petiole necrosis at 8 and 16 dpi. The presence of a whitish mycelium in the necrotic leaf area was also evident. 

**Figure 3:**
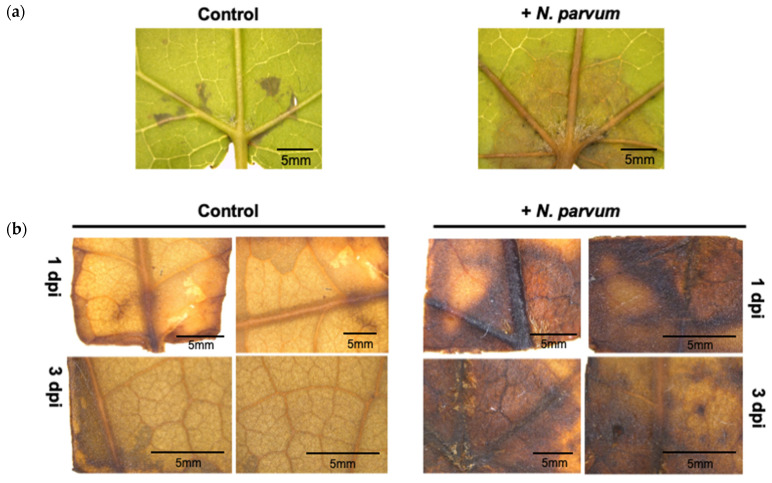
DAB staining of *L. styraciflua* leaves infected with *N. parvum* at early stages of infection. (**a**) Symptoms in leaves at 3 dpi. (**b**) V H_2_O_2_ detection by DAB staining in infected and non-infected (control) leaves. Representative images of DAB stained leaves are shown. A stereomicroscope was used to acquire the images.

Since it was evident that the disease symptoms appear at early stages, diaminobenzidine (DAB) staining to indirectly detect the hydrogen peroxide production in *L. styraciflua* leaves at 1 and 3 dpi was used. The presence of a dark brown precipitate was detected in the infected leaves at early time points (1 and 3 dpi), indicating the presence of H_2_O_2_ (Figure 3 and Figure S3).

**Figure 4:**
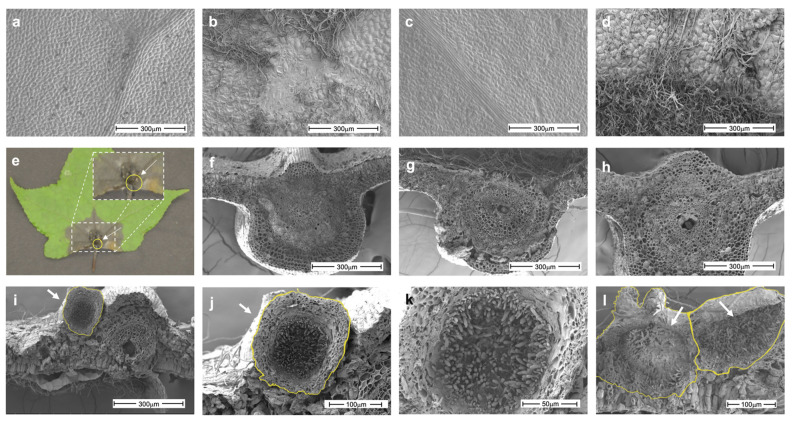
Analysis by scanning electron microscopy of *L. styraciflua* leaves infected with *N. parvum*. (**a**,**c**) Control adaxial leaves at 3 and 8 dpi, respectively. (**b**,**d**) Infected axial leaves at 3 and 8 dpi, respectively. (**e**) Leaf base infection: the dashed squares indicate the zones that were analyzed in (**f**–**l**). (**f**,**h**) Control at 3 and 8 dpi, respectively. (**g**) Infected sample at 3 dpi. (**i**–**l**) Infected sample at 8 dpi. (**j**,**k**) Close-ups of panel (**i**). The pycnidia are delimited with a solid yellow line. Arrows indicate the pycnidium and conidia of *N. parvum.*

The SEM images revealed that the fungus grew robustly on the leaf (adaxial) surface, forming a hyphal mass and causing tissue degradation in which the cuticle and wax integrity were compromised (Figure 4a–d). In addition, the infection provoked petiole degradation. A transverse cut of the leaf base showed that the fungus developed pycnidia, asexual reproductive structures. These pycnidia appeared individually or as aggregates embedded in the plant tissue with thick walls (Figure 4i–l). A longitudinal section of pycnidium showed mature conidia. The conidiogenic oval cells were presented perpendicular to the walls of the pycnidium.

**Figure 5:**
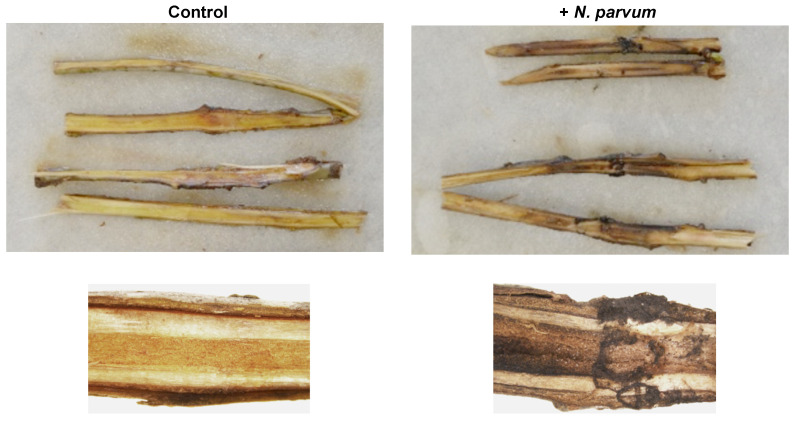
Infection of *L. styraciflua* fresh stems with *N. parvum*. The photographs were taken at 7 dpi.

Finally, since the *Botryosphaeriaceae* family members are characterized as woody-plant pathogens, the pathogenicity of the Liqui 1–3 strain on fresh stems of *L. styraciflua* was tested. As shown, *N. parvum* at 7 dpi triggered distinct symptoms of disease, including discoloration and necrosis, covering a zone beyond the site of inoculation (Figure 5).

### 3.3. Identification and Description of CysRPs in L. styraciflua and N. parvum 

CysRPs have been widely studied for their important functions in plant–pathogen interactions, in both the host and pathogen. To identify CysRPs in *L. styraciflua* and *N. parvum*, two databases with transcriptomic and genomic information were analyzed. For each organism, five sequences encoding CysRPs were identified (named as LsCysRP1-5 and NpCysRP1-5). All of these contained a putative start and stop codon, apart from the LsCysRP3 sequence, in which a stop codon was absent (Table 1 and Table S2). The amino acid sequence length varied between 95 and 204, and the molecular weight ranged from 7.7 to 17.5 kDa. Interestingly, with the exception of LsCysRP1, which had a calculated isoelectric point (pI) of 6.04, the calculated pI values of all of the LsCysRPs exceeded 8.67. In contrast, apart from NpCysRP1 with a pI of 7.57, the calculated pI values of all the NpCysRPs were lower than 5.48 (Table 1). To predict the disulfide bonds *in L. styraciflua* and *N. parvum* CysRPs, the servers Cyscon, Disulfind, DiANNA, CYS_REC, and SCRATCH were used. The results revealed that LsCysRP1, LsCysRP2, LsCysRP3, LsCysRP4, and LsCysRP5 have the potential to form 3, 4, 4, 0, and 6 disulfide bonds, respectively. Meanwhile, in *N. parvum*, 6, 4, 4, 5, and 4 disulfide bridges were estimated for NpCysRP1, NpCysRP2, NpCysRP3, NpCysRP4, and NpCysRP5, respectively. The connectivity pattern of the cysteine residues forming the disulfide bonds varied between prediction methods (Table S3). To determine whether the CysRPs have the potential for secretion, an analysis was conducted with the TargerP-2.0 server. All sequences, apart from the LysCysRP2 sequence, have a peptide signal between 17 and 27 amino acids. To corroborate these results, additional analyses were performed using the Protter and DeepLoc-1.0 servers, and the results indicated that all of the CysRPs have a signal peptide and are extracellular proteins (Table 2).

To identify possible functions and regions of similarity in the CysRPs, BLAST, and MOTIF tools were used. No significant similarity was found for LsCysRP1, 2, and 4, or for NpCysRP1, 2, and 3. In contrast, LsCysRP3 showed similarity with an LTP and LsCysRP5 with a gibberellin-regulated protein 1-like protein. Interestingly, a CFEM domain was identified for both NpCysRP4 and 5 (Table 1).

### 3.4. CysRP Phylogenetic Analyses

**Figure 6:**
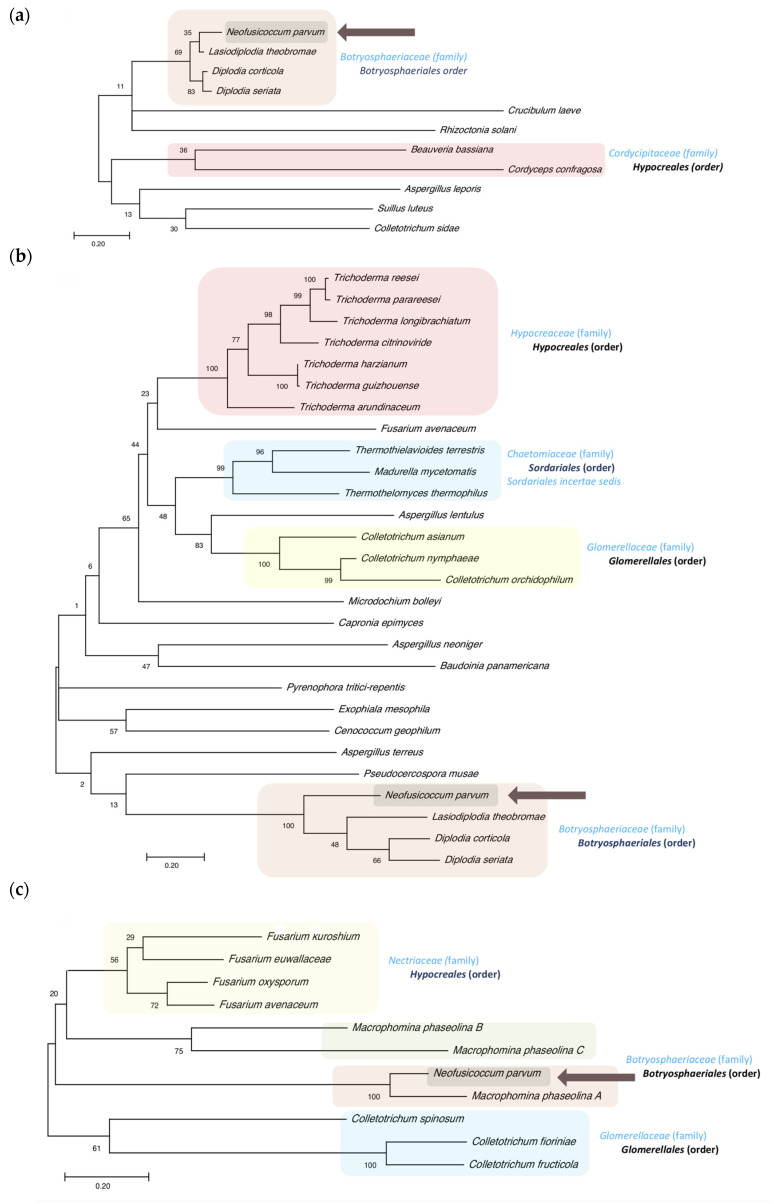
Phylogenetic analyses of NpCysRP1, NpCysRP2, and NpCysRP3 with orthologs from other fungal species. The phylogenetic trees were constructed using protein sequences with the maximum likelihood method and JTT matrix-based model. NpCysRP1, NpCysRP2, and NpCysRP3 trees with the highest log likelihood values, (−2656.73), (−15,145.13), and (−5228.55), respectively, are shown. The percentage of trees in which the associated taxa clustered together is shown next to the branches. The trees are drawn to scale, with branch lengths measured in terms of the number of substitutions per site. (**a**) For NpCysRP1, the amino acid sequences are from *N. parvum* (EOD50423.1), *L. theobromae* (KAB2569989.1), *D. corticola* (XP_020126718.1), *D. seriata* (KKY27788.1), *C. laeve* (TFK32468.1), *R. solani* (CUA75287.1), *B. bassiana* (PQK10211.1), *C. confragosa* (OAA70963.1), *A. leporis* (KAB8069877.1), *S. luteus* (KIK46160.1), and *C. sidae* (TEA07484.1). (**b**) For NpCysRP2, the amino acid sequences are from *T. reesei* (XP_006969324.1), *T. parareesei* (A9Z42_0055550), *T. longibrachiatum* (PTB72285.1), *T. citrinoviride* (XP_024746670.1), *T. harzianum* (XP_024771023.1), *T. guizhouense* (PB44849.1), *T. arundinaceum* (RFU76343.1), *F. avenaceum* (KIL85060.1), *T. terrestris* (XP_003656631.1) *M. mycetomatis* (KXX77126.1), *T. thermophilus* (XP_003666291.1), *A. lentulus* (KKO98531.1), *C. asianum* (KAF0319766.1), *C. nymphaeae* (KXH38321.1), *C. orchidophilum* (XP_022481280.1), *M. bolleyi* (KXJ94843.1), *C. epimyces* (XP_007733601.1), *A. neoniger* (XP_025474954.1), *B. panamericana* (XP_007680904.1), *P. tritici-repentis* (XP_001934627.1), *E. mesophila* (RVX68508.1), *A. terreus* (XP_001217394.1), *P. musae* (KXT13283.1), *N. parvum* (EOD50922.1), *L. theobromae* (KAB2578904.1), *D. corticola* (XP_020125849.1), and *D. seriata* (KKY21900.1). (**c**) For NpCysRP3, the amino acid sequences are from *F. kuroshium* (RMI89680.1), *F. euwallaceae* (RTE68176.1), *F. oxysporum* (EXK86396.1), *F. avenaceum* (KIL83641.1), *M. phaseolina B*, (EKG22346.1), *M. phaseolina C* (EKG22347.1), *N. parvum* (EOD52838.1), *M. phaseolina* A (EKG20686.1), *C. spinosum* (TDZ33147), *C. fioriniae* (EXF77634.1), and *C. fructicola* (XP_031888108).

**Figure 7:**
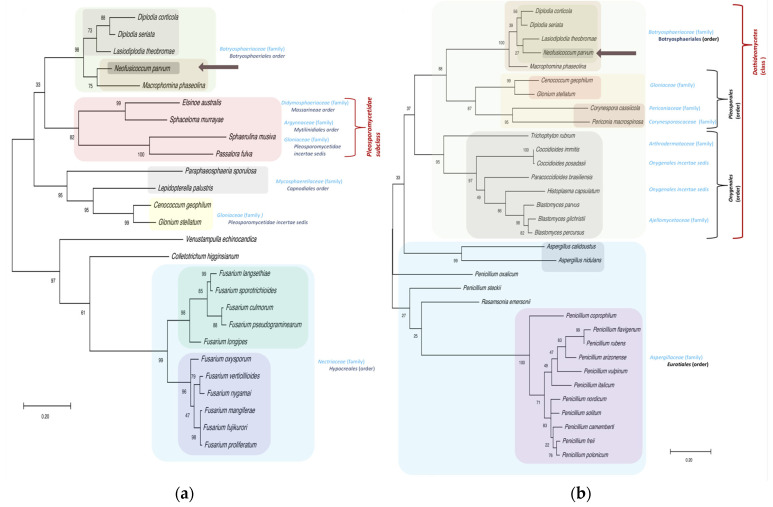
Phylogenetic analyses of NpCysRP4 and NpCysRP5 with orthologs from other fungal species. The phylogenetic trees were constructed using protein sequences with the maximum likelihood method and JTT matrix-based model. NpCysRP4 and NpCysRP5 trees with the highest log likelihood values, (−4619.05) and (−6976.31), respectively, are shown. The percentage of trees in which the associated taxa clustered together is shown next to the branches. The trees are drawn to scale, with branch lengths measured in terms of the number of substitutions per site. (**a**) For NpCysRP4, the amino acid sequences are from *D. corticola* (XP_020129061.1), *D. seriata* (KKY13873.1), *L. theobromae* (KAB2571564.1), *N. parvum* (EOD51105.1), *M. phaseolina* (EKG14570.1), *E. australis* (TKX25331.1), *S. murrayae* (PNS17704.1), *S. musiva* (XP_016758053.1), *P. fulva* (AQA29231.1), *P. sporulosa* (XP_018029092.1), *L. palustris* (OCK84136.1), *C. geophilum* (OCK98732.1), *G. stellatum* (OCL13334.1), *V*. *echinocandica* (RDL36609.1), *C. higginsianum* (TIC93879.1) *F. langsethiae* (KPA46759.1), *F. sporotrichioides* (RGP70600.1), *F. culmorum* (PTD11477.1), *F. pseudograminearum* (XP_009256009.1), *F. longipes* (RGP60032.1), *F. oxysporum* (EXA53032.1), *F. verticillioides* (RBR06989.1), *F. nygamai* (PNP52701.1), *F. mangiferae* (CVK83605.1), *F. fujikurori* (QGI58805.1), and *F. proliferatum* (XP_031075444.1). (**b**) For NpCysRP5, the amino acid sequences are from *D. corticola* (XP_020133845.1), *D. seriata* (KKY24076.1), *L. theobromae* (KAB2577423.1), *N. parvum* (EOD44996.1), *M. phaseolina* (EKG19367.1), *C. geophilum* (OCK97775.1), *G. stellatum* (OCL07361.1), *C. cassiicola* (PSN73263.1), *P. macrospinosa* (PVI08476.1), *Trichophyton rubrum* (XP_003231199.1), *C. immitis* (XP_001240075.1), *C. posadasii* (XP_003069153.1), *P.s brasiliensis* (ODH22777.1), *H. capsulatum* (EEH05575.1), *Bl. parvus* (OJD22636.1), *A. calidoustus* (CEL09455.1), *A. nidulans* (XP_660049.1), *P. oxalicum* (EPS32950.1), *P. steckii* (OQE27319.1), *Rasamsonia emersonii* (XP_013330685.1) *P. coprophilum* (OQE36870.1) *P. flavigenum* (OQE32686.1), *P. rubens* (XP_002567673.1), *P. arizonense* (XP_022485186.1), *P. vulpinum* (OQE12392.1), *P. italicum* (KGO74509.1), *P. nordicum* (KOS37798.1), *P. solitum* (OQD85155.1), *P. camemberti* (CRL28928.1), *P. freii* (KUM58798.1), and *P. polonicum* (OQD69393.1).

To obtain more information about the NpCysRPs, a phylogenetic analysis was conducted for each of these proteins (Figure 6 and Figure 7). All of the NpCysRPs were clearly grouped in the *Botryosphaeria* lineage, including *Lasiodiplodia, Diplodia*, and *Macrophomina* species. However, the species in the subclade were not always the same. NpCysRP1, 2 and 5 were closely related to *Lasiodiplodia theobromae*, while NpCysRP3 and 4 shared a branch with *Macrophomina phaseolina*.

The NpCysRP1 phylogeny revealed the existence of few orthologous sequences for this protein in databases, and this protein occurred in the *Botryosphaeriaceae* family, only in the species *L. theobromae* (85.00% identity), *Diplodia corticola* (85.42% identity) and *Diplodia seriata* (82.29%, identity), as well as in the family *Cordycipitaceae* of the order *Hypocreales*, characterized by entomopathogenic fungal species, such as *Beauveria bassiana* and *Cordyceps confragosa*, but with low identities (29.67% and 31.36%, respectively). However, the cysteines remained at the same site in the sequences (Figure S4). Other species, such as *Aspergillus leporis* and *Rhizoctonia solani*, were included in the alignment; however, these sequences had a lower number of cysteines and shared lower identity.

In addition to the *Botryosphaeria* lineage, the NpCysRP2 phylogenetic tree contained orthologs of the family *Hypocreaceae*, order *Hypocreales*, including seven different species of the *Trichoderma* genus with identities that ranged from 33.04 to 38.18%. There were also members of the family *Glomerellaceae* present, including species of the well-known phytopathogenic genus *Colletotrichum*, such as *Colletotrichum asianum*, *Colletotrichum nymphaeae*, and *Colletotrichum orchidophillum*, with 33.04, 36.28, and 37.19% amino acid identity, respectively. Interestingly, the tree also included the human pathogen *Madurella mycetomatis* of the order *Sordariales*, with 33.90% identity. The alignment revealed that NpCysRP2 introduces a new cysteine-rich domain with the consensus motif C1[Y/F]xPx9-10C2x6-8C3C4x4C5x2Nx2C6x10-23C7Tx8C9x3C10 at the N-terminus. Moreover, the multiple-sequence alignment of NpCysRP2 with the ortholog sequences of *L. theobromae* (67.52% identity) and *D. corticola* (62.07% identity) showed that NpCysRP2 is a larger protein, since the *L. theobromae* and *D. corticola* sequences contain 310 and 296 amino acids, respectively (Figure S4). The proteoforms of *L. theobromae* and *D. corticola* presented a transmembrane domain crossing at the carboxyl-end, while the predicted proteoforms for all of the sequences used in the phylogenetic analysis (26 in total) showed the transmembrane helix (Figure S5). A particular phylogeny was noted in the case of NpCysRP3 (Figure 6C); this protein did not present a well-defined clade distribution within the *Botryosphaeriaceae* family, and only the *M. phaseolina* orthologs (MpCysRPs A, B, and C) were found, with 64.46, 29.17 and 30.83% identity, respectively. The *Nectriaceae* family was represented in the phylogenetic tree with some members of the unique and fascinating Ambrosia *Fusarium* clade, represented by *Fusarium euwallaceae* and *Fusarium kuroshium*, which has been recognized recently as an emerging fungal pathogen [54,55]. NpCysRP5 has a CFEM domain characterized by eight cysteines with the specific consensus motif PxC^1^[A/G]x_2_C^2^x_8_-12C^3^x_1-3_[x/T]Dx_2-5_C^4^xC^5^x_9-14_**C**^6^x_3-4_C^7^x_15-16_C^8^ [56] and where P62 (in *N. parvum* sequence) was conserved in this group, in addition to the conserved amino acids of the CFEM motif (Figure S6). NpCysRP4 and its orthologs have a CFEM domain (we refer to this as a CFEM-like domain) with a conserved extra cysteine (C58 in the *N. parvum* sequence). Outside the CFEM domain, another cysteine (C94 in *N. parvum*) was conserved, forming the consensus motif PxC^1^[A/G]x_2_C^2^x_8-12_C^3^x_1-3_[x/T]Dx_2-5_C^4^xC^5^x_8-13_**C**^6^C^7^x_3-4_C^8^x_15-16_C^9^x_12-13_**C**^10^. NpCysRP4 and NpCysRP5 are well represented in the clade of the *Botryosphaeriaceae* family, but NpCysRP4 shares a subclade with *M. phaseolina*, with 78.07% identity, while NpCysRP5 presents 83.01% identity with *L. theobromae*, the species *Cenococcum geophilum* and *Glonium stellatum* belong to the *Gloniaceae* family and present orthologs for both NpCysRP4 and NpCysRP5. Various orthologs were identified for NpCysRP4 in the *Nectriaceae* family, including in the order *Hypocreales* represented by the well-known phytopathogenic *Fusarium* species *Fusarium verticillioides* and *Fusarium oxysporum*, which were not found in the NpCysRP5 phylogenetic tree using 33 sequences. In the case of NpCysRP5, a clade represented by the order *Eurotiales* was clearly identified, grouping different *Penicillium* species with identities that ranged from 34.3 to 41.78%.

In the case of the *L. styraciflua* CysRPs, no similar sequences were found for LsCysRP1, 2 and 4, and it was therefore impossible to construct the corresponding phylogenetic trees. For LsCysRP3, the BLAST^®^ result revealed a sequence identity of 66.98–64.15% with various *Gossypium* species and 63.81% with *Vitis pseudoreticulata*. Finally, LsCysRP5 showed 73.83–65.42% identity with different *Quercus* species, 74.77% identity with *Castanea mollissima*, and 67.29% identity with *Durio zibethinus*.

### 3.5. Expression of CysRP mRNAs of L. styraciflua and N. parvum during Early Stages of the Infection Process

**Figure 8:**
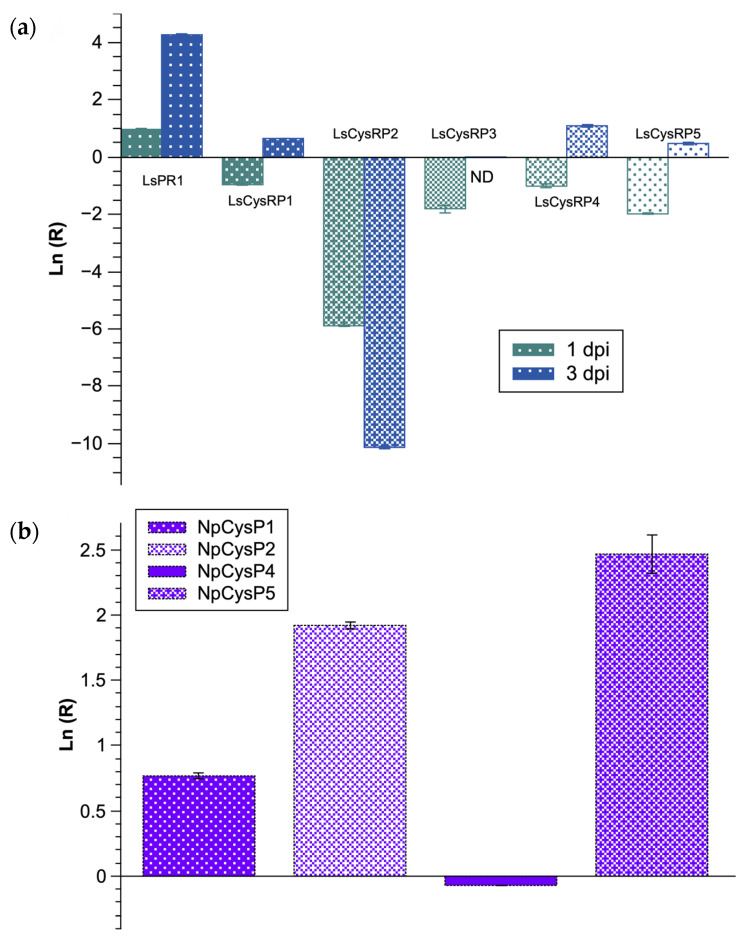
Expression in the early stages of infection of CysRP mRNAs of both *L. styraciflua* and *N. parvum*. (**a**) LsCysRP mRNA expression at 1 and 3 dpi, RNA was extracted from leaves with mechanically injury and used as a control. The geometric mean between Tip41 and actin was used as a reference. For all of the transcript statistics, significant differences were detected between 1 and 3 dpi at *p* < 0.01 using Student’s *t* test. (**b**) NpCysRP mRNA expression at 3 dpi, 1 dpi was used as control. For the analysis, NpCysRP3 was used as a reference gene. The data are the average of three independent inoculation experiments.

To explore whether the transcription of CysRP mRNAs of *L. styraciflua* and *N. parvum* was modulated at early stages of the interaction, quantitative polymerase chain reaction (qPCR) tests were performed (Figure 8). LsPR1, an ortholog of the *Nicotiana tabacum* gene encoding pathogenesis-related protein 1 (PR1), a protein involved in the defense response in plants and usually used as a defense marker. The qPCR results showed an increase in LsPR1 mRNA content at 1 and 3 dpi. The opposite profile was observed for all LsCysRP transcripts, since the expression decreased after 1 dpi, and LsCysRP2 presented the lowest level at this time post-infection. At 3 dpi, LsCysRP2 presented the most significant change, and LsCysRP3 remained unchanged, while LsCysRP1, 4, and 5 presented a mild increase (Figure 8a).

Finally, the experimental design allowed comparing the expression of NpCysRP transcripts between 1 and 3 dpi. There was an increase in the expression of NpCysRP1, 2, and 5 mRNAs, with NpCysRP5 presenting the most significant increase (Figure 8b). In contrast, NpCysRP4 showed a significant decrease.

## 4. Discussion

*Liquidambar styraciflua* L. (*Altingiaceae*) is used for reforestation and landscaping and has been proposed as an attractive hardwood species for potential bioenergy production [23]. Given its importance, it is important to determine the factors that affect the integrity of this tree, i.e., phytosanitary problems. It is generally assumed that *L. styraciflua* is associated with certain pathogens [57]. In this context, information collected by Hepting in 1971 [58] listed *Cercospora liquidambaris*, *Septoria liquidambaris*, *Exosporium liquidambaris*, *Leptothyriella liquidambaris,* and *Gloesporium nervisequm* as common foliar pathogens of the sweetgum. On the other hand, *L. theobromae* and *Botryosphaeria dothidea* have been identified as responsible for provoking stem cankers and dieback in seedlings located in nurseries and outplantings in the USA [57,58]. Currently, there is no new information about the nature of pathogenic fungi infecting *L. styraciflua*, but there is a prevalence of the incidence of symptoms associated with diseases. Here, we report that *N. parvum* is associated with foliar damage in *L. styraciflua*. The pathosystem established in this study identified Liqui 1−3 as a very aggressive strain, since it provokes several symptoms at early stages post infection (Figure 1 and Figure 2). The SEM analysis showed that the fungus was able to grow and develop pycnidia to produce a large number of mature spores, which acts to increase its infective potential (Figure 4). Our results are consistent with those reported for other *Botryosphaeria* species. Amponsah et al. [59] reported that, during the interaction of grapevine and *N. luteum*, conidial germination occurred at a faster rate (3 h after inoculation) on detached and wounded leaf and shoot surfaces than on attached and non-wounded leaf surfaces, indicating that conidium adhesion, germination and development were affected by the condition of the host plants. Moreover, in mamey sapote stem cuttings infected with *L. theobromae* at 30 dpi, it was possible to identify fruiting bodies embedded in the host tissue [60]. It will be interesting in future studies to characterize the Liqui 1−3 strain in greater detail, with respect to its capacity to produce lytic enzymes and secondary metabolites with phytotoxic effects in comparison to other strains isolated from other ecological niches. Finally, attention should also be given to other fungi isolated from *L. styraciflua* with pathogenic potential, such as Liqui 1–02 and Liqui 2–2 (Figure 1 and Figure S1).

In addition to the phenotypic effects associated with the pathogenicity caused by *N. parvum* in *L. styraciflua*, the identification of CysRPs was considered part of the molecular events triggered by both organisms during infection and defense response. Five CysRPs were identified for each organism (Table 1) and bioinformatic analyses revealed that LsCysRP1, 2, and 4 are proteins with unknown functions, suggesting that they are species-specific. LsCysRP3 was recognized as an LTP. This protein is considered an antifungal protein classified as PR-14 [61] with yet unknown mode of action, but in vitro conditions have the ability to enhance intermembrane exchange, causing *a posteriori* fungal cell death [62]. In barley leaves, LsCysRP3 inhibits the growth of *Fusarium solani* [63], while transgenic *Populus tomentosa* overexpressing an LTP of *Leonurus japonicus* is resistant to *Alternaria alternata* and *Colletotrichum gloeosporioides* [64]. LsCysRP5 was identified as a gibberellin-regulated protein 1-like protein. These proteins are named the GASA family in *A. thaliana* and Snakins in *Solanum tuberosum*. This class of proteins forms 5 or 6 disulfide bonds necessary for structure as a consequence of protein function [65]. Moreover, the overexpression of Snakin protein 1 in potato enhances resistance to relevant pathogens [66]. The Snakin-2 of French bean may form a 42-kDa protein complex with a proline-rich protein and could participate in the plant defense process [67]. Thus, in comparison with the reported orthologs of LsCysRP3 and 5, these *L. styraciflua* proteins may present antifungal activity. The expression patterns of the respective mRNAs of LsCysRP3 and 5 during the interaction with *N. parvum* are particularly interesting because of the repressive behavior profile of both mRNAs (Figure 8). 

It is well known that many pathogens have evolved tools to evade the immune system of the host, so it will be interesting to investigate this effect further in future studies. Through genomic and transcriptomic analyses, it has already been recognized that the pathogenic and virulent protein arsenal of *N. parvum* potentially comprises enzymes that facilitate wood degradation and host colonization [68,69]. To the best of our knowledge, our research is the first to identify new CysRPs with potential roles in pathogenesis. Following examination of the genomic information of *N. parvum*, five sequences with high cysteine content were selected, and the analysis identified NpCysRP5 as a CFEM domain protein, distinguishable from other cysteine-rich domains and inherent to fungi [56]. Although this class of motifs is well represented in the *Ascomycota* phylum, pioneering research on the CFEM domain [56,70] did not include members of the *Botryosphaeriaceae* family. In this sense, NpCysRP5 is identified for the first time as an orthodox CFEM protein of *N. parvum* that is expressed early during the infection process in *Liquidambar*. Interestingly, NpCysRP4 (a protein that apparently lacks a transmembrane domain) has a CFEM-like domain with two additional well-conserved cysteines (Figure S6). The presence of these cysteines in NpCysRP4 could cause a disulfide rearrangement and increase conformational stability [71,72], and could increase the possibility of creating additional proteoforms with complementary functions, contributing to virulence. In accordance with the report by Zhang et al. [56], a positive correlation between CFEM domain occurrence and fungal pathogenicity was shown, and it seems that the occurrence of CFEM domain proteins is independent of the lifestyle of pathogen (i.e., biotrophic, hemibiotrophic, or necrotrophic). For example, in *Botrytis cinerea* (a necrotrophic fungus), BcCFEM1 is highly expressed at early stages of infection in *Phaseolus vulgaris*, and gene disruption acts to decrease virulence [73]. For *Magnaporthe grisea*, a hemibiotrophic filamentous ascomycete, the mutant pth11 (mutated in a CFEM transmembrane protein) is impaired during appressorium maturation, which influences infection capacity [74]. However, it is important to note that not all CFEM proteins play a role in pathogenicity. For example, three CFEM motif GPI-anchored proteins from *Aspergillus fumigatus* participate in cell wall stability but not in fungal virulence [75]. Here, we identified a CFEM motif-containing protein of *N. parvum* (NpCysRP5) that also contains a putative transmembrane helix, and is expressed during early infection stages in *L. styraciflua*. It will be of great interest to elucidate further the exact contribution of this protein in pathogenesis. The phylogenetic analyses identified orthologs of NpCysRP4 and NpCysRP5 in additional *Botryosphaeriaceae* species; thus, contributing to the knowledge of this important pathogen of woody plants (Figure 7). Moreover, the expression analysis indicated contrasting mRNA expression profiles for NpCysRP4 and NpCysRP5 (Figure 8). Since only the NpCysRP5 mRNA expression showed a positive correlation during early stages of infection, we believe that NpCysRP5 is a better candidate with a significant role during pathogenesis, highlighting the fact that NpCysRP4, with a CFEM-like domain, has another important function that could be identified in further study. NpCysRP2 also introduces a new cysteine-rich domain with the following characteristics: (1) the deduced consensus motif is C1[Y/F]xPx9-10C2x6-8C3C4x4C5x2Nx2C6x10-23C7Tx8C9x3C10; (2) all cysteines are conserved; and (3) a transmembrane helix toward the carboxyl terminus is also conserved. All of these data indicate that NpCysRP2 is a new protein, which we refer to as a fungal cysteine-rich transmembrane protein (FCRTP). Finally, the phylogenetic analyses highlighted two important points: (1) NpCysRPs are present in various fungal families, and (2) each NpCysRP is associated with a particular species. For example, for NpCysRP4, the order *Eurotiales* is well represented by several *Penicillium* species, while for NpCysRP5, the order Hypocreales is represented by members of the *Fusarium* genus, indicating that *N. parvum* is a very versatile fungus.

## 5. Conclusions

In this study, we showed that *N. parvum* is an aggressive pathogen of *L. styraciflua*. We also identified novel CysRPs of both interacting organisms, such as NpCysRP2, which seems to be conserved in various fungi and contains a novel cysteine-rich motif and a putative transmembrane domain. In addition, NpCysRP4 was identified as a novel CFEM-like domain. In the case of LsCysRPs, three of the identified proteins did not play a predicted putative function. Analysis of expression during the plant–pathogen interaction revealed that all of the LsCysRPs presented a repressive expression profile in comparison with that of NpCysRPs, suggesting the involvement of an interesting molecular mechanism during this interaction. Finally, all of the CysRPs identified in this study are suitable candidates for further investigation in order to increase the knowledge regarding the molecular processes that occur during the defense response of woody species to by *N. parvum* infection.

## Figures and Tables

**Table 1:** Amino acid sequences of *L. styraciflua* and *N. parvum* CysRP. Predicted putative function was inferred with BLAST and Motif prediction tools, molecular weight, and isoelectric point are theoretical predictions (see Materials and Methods). Cysteines are underlined in the amino acid sequences.

Assigned Name	Target Name	Protein	Length	Total Number of Cysteines	Molecular Weight (kDa)	pI	Predicted Putative Function
LsCysRP1	comp30850_c0_seq1	MDRLSSMRSVFWILVSFGTNAAVSELINVACPQSAEVDHCMCNCRAIDDAWNVDWHNDLRMFGLKTEIQRPQNEVCCNLVTSCVFIHWDLVSHRKSRNLR	100	7	8.9	6.04	No significant similarity found
LsCysRP2	comp28855_c0_seq1	MGMALFCPVSGLQQQITAATSKFDGKGTSGVGACCTKPCPCQHQMKKLCCISSYGYCNRFRFKELSYHHLVAKANHSWLSCIEVIFAIPYSSADLGWLEMVKP	103	9	9.5	8.84	No significant similarity found
LsCysRP3	comp18453_c1_seq1	MASSGILNLACVVLVSMVLVAPHAEAAMTCGQVTSSVSPCLPYLKGASPLQPGCCNGIRSLNSAAKTTPDRQAACRCLQQAASSISGINLGLASGLPGKCGISI	104	8	7.7	9.03	Lipid-transfer protein type 1
LsCysRP4	comp37785_c0_seq1	MGFTGMSPMSFLCAWCCGQPTHHLEALVSWTAALLQSLKLKVAEDTDFFTNSFDPVEVWIISLYKYLYHKTLRKTNIGKEAALKRAKPMMIACVVEMLLIDLQS	104	4	8.9	8.8	No significant similarity found
LsCysRP5	comp31840_c0_seq3	MAIYKALLASLLVSLFVLHLVVADGMGMQTTDAATSSPPKSSPPKKIDCGGACSSRCQLSSRPNLCKRACGTCCARCNCVPPGTSGNEDVCPCYASMTTHGGRHKCP	107	12	8.6	8.67	Gibberellin-regulated protein 1-like
NpCysRP1	EOD50423.1	MQFKFALVSLFTALALAAPGIDLEKRCTANGGSCTQLSQCCSGNCEYDSSIGLVCKAASKKEKRCTANGGACTQLAQCCSGNCEYDSSIGLVCKP	95	12	7.8	7.57	Hypothetical protein UCRNP2_2803
NpCysRP2	EOD50922.1	MRPLYSHLVHTLPPLFSILLDPRPASAQDDHVPRCYYPDGTLASNDYACRLNTTESFCCTTNVTCLDNKICQVLAPTQYEYNRGTCTDKTWTSDECPKFCQAQSPSYGSGVIRPVY	116	9	10	4.79	Hypothetical protein UCRNP2_2308
NpCysRP3	EOD52838.1	MRFFVAISVAATALFSTGLAASHAKCACQIASDRGTNDAATAGMCSYVGGVMSKSNVAFEKQVATWSLTSILADNTITTGVLYPGKYCQGIGGHELGGDAVYEACRSQKCGDTYCQDSTCV	121	8	10.5	5.48	Hypothetical protein UCRNP2_338
NpCysRP4	EOD51105	MRVSAFAAILGYSSLALAQTQLLPTCAQKCVGTDFGGCSTVDVKCICANKELLTGLACCVSTGCNAADQETVINFAQNLCKPQGVTDLPTTATCASGASSATSSASSGTASTTASSAASASASATGSAASSASSAASSVASSAASAASSAAASASAATTAGSGAGSCQGNAAGMGFGLVLAGLLGAL	187	11	15.5	4.53	Putative CFEM domain protein
NpCysRP5	EOD44996.1	MKTSFAAVAFSLASVAYSQNISDLPSCSLNCFVTTLGSDGCSSLTDFECHCKVPGLTDEITPCVQKACSAADQQTVAEQVQALCAQYGVSISVPEASSTAAPSSTAAATSAPASSSAAATTAASSAASSAASSASASATSTETASPSATDVVISTPVATPTTPGASTPAGSTPTPSQFQGAAGKTGVVGGVVGFAAAVAAVAAL	204	8	17.6	4.02	Putative CFEM domain protein

**Table 2:** Predicted cellular location of *L. styraciflua* and *N. parvum* CysRPs.

Assigned Name	TargetP-2.0 Server Prediction	Cleavage Site between Pos	Likelihood	Protter	Cleavage Site between Pos	DeepLoc-1.0 Prediction	Likelihood	Soluble/Membrane
LsCysP1	Signal peptide	24–25	0.9931	Signal peptide	21–22	Extracellular	0.8321	0.9813/0.0187
LsCysP2	Other	⎻⎻⎻⎻	0.7782	Signal peptide	18–19	Extracellular	0.8088	0.9946/0.0054
LsCysP3	Signal peptide	26–27	1	Signal peptide	26–27	Extracellular	0.9998	1/0
LsCysP4	Signal peptide	26–27	0.7135	Signal peptide	26–27	Extracellular	0.7316	0.9415/0.0585
LsCysP5	Signal peptide	23–24	1	Signal peptide	23–24	Extracellular	0.9973	1/0
NpCysP1	Signal peptide	17–18	1	Signal peptide	20–21	Extracellular	1	1/0
NpCysP2	Signal peptide	27–28	0.9962	Signal peptide	27–28	Extracellular	0.8773	0.9997/0.0003
NpCysP3	Signal peptide	20–21	1	Signal peptide	20–21	Extracellular	0.999	0.998/0.002
NpCysP4	Signal peptide	18–19	0.999	Signal peptide	18–19	cell membrane/extracellular/Endoplasmic reticulum	0.2925/0.2815 /0.2539	0.3246/0.6754
NpCysP5	Signal peptide	18–19	0.9986	Signal peptide	18–19	Extracellular/Endoplasmic reticulum/Cell membrane	0.3539/0.2639 /.2186	0.4355/0.5645

## Data Availability

Pre-print version (doi:10.21203/rs.3.rs-26140/v3).

## Supplementary Materials

The following are available online at https://www.mdpi.com/article/10.3390/jof7121027/s1, Figure S1: Pathogenicity screening in A. thaliana seedlings. Figure S2: Molecular identification of the Liqui 1−3 strain, Figure S3: Detection of H2O2 by DAB staining of L. styraciflua leaves infected with N. parvum at early stages of infection. A stereomicroscope was used to acquire the images, Figure S4: Multiple-sequence alignment of NpCysRPs and other orthologous sequences. A. NpCysRP1, B. NpCysRP2, and C. NpCysRP3. Figure S5: Predicted proteoforms of NpCysRP2 and other orthologous sequences, Figure S6: Multiple-sequence alignment of NpCysRPs and other orthologous sequences. A. NpCysRP4, B. NpCysRP5. Table S1: Primers used in this study for the molecular identification of Liqui 1−3 strain and qRT-PCR experiments, Table S2: CysRP nucleotide sequences, Table S3: Predicted disulfide bonds for CysRPs.

## References

[1] Slippers B., Wingfield M.J. (2007). Botryosphaeriaceae as endophytes and latent pathogens of woody plants: Diversity, ecology and impact. Fungal Biol. Rev..

[2] Sakalidis M.L., Slippers B., Wingfield B.D., Hardy G.E.S.J., Burgess T.I. (2013). The challenge of understanding the origin, pathways and extent of fungal invasions: Global populations of the Neofusicoccum parvum-N. ribisspecies complex. Divers. Distrib..

[3] Lorenzini M., Cappello M.S., Zapparoli G. (2015). Isolation of Neofusicoccum parvum from withered grapes: Strain characterization, pathogenicity and its detrimental effects on passito wine aroma. J. Appl. Microbiol..

[4] Carrillo J., Eskalen A., Rooney-Latham S., Scheck H.J. (2016). First Report of Neofusicoccum nonquaesitum Causing Branch Canker and Dieback of Avocado in California. Plant Dis..

[5] Molina-Gayosso E., Silva-Rojas H.V., García-Morales S., Avila-Quezada G. (2012). First Report of Black Spots on Avocado Fruit Caused by Neofusicoccum parvum in Mexico. Plant Dis..

[6] Valencia A.L., Gil P.M., Latorre B.A., Rosales I.M. (2019). Characterization and Pathogenicity of Botryosphaeriaceae Species Obtained from Avocado Trees with Branch Canker and Dieback and from Avocado Fruit with Stem End Rot in Chile. Plant Dis..

[7] Espinoza J.G., Briceño E.X., Chávez E.R., Úrbez-Torres J.R., Latorre B.A. (2009). Neofusicoccum spp. Associated with Stem Canker and Dieback of Blueberry in Chile. Plant Dis..

[8] Palavouzis S., Tzamos S., Paplomatas E., Thomidis T. (2015). First report of Neofusicoccum parvum causing shoot blight of pomegranate in Northern Greece. New Dis. Rep..

[9] Song Z.X., Liao J., Luo H., Zhang F., Sun Z.X., Liu Q.K., Deng J.X. (2019). First Report of Neofusicoccum parvum Associated with Shoot Blight on Peaches in China. Plant Dis..

[10] Cheon W., Kim Y.S., Lee S.G., Jeon Y.H., Chun I.-J. (2013). First Report of Branch Dieback of Walnut Caused by Neofusicoccum parvum in Korea. Plant Dis..

[11] Aćimović S.G., Rooney-Latham S., Albu S., Grosman D.M., Doccola J.J. (2018). Characterization and Pathogenicity of Botryosphaeriaceae Fungi Associated with Declining Urban Stands of Coast Redwood in California. Plant Dis..

[12] Golzar H., Burgess T.I. (2011). Neofusicoccum parvum, a causal agent associated with cankers and decline of Norfolk Island pine in Australia. Australas. Plant Pathol..

[13] Iturritxa E., Slippers B., Mesanza N., Wingfield M.J. (2011). First report of Neofusicoccum parvum causing canker and die-back of Eucalyptus in Spain. Australas. Plant Dis. Notes.

[14] Lopes A. (2016). Diversity and phylogeny of Neofusicoccum species occurring in forest and urban environments in Portugal. Mycosphere.

[15] Mirhosseini H., Babaeizad V., Rahimlou S. (2014). Neofusicoccum parvum, agent of leaf spot on the new host Ginkgo biloba in Iran. New Dis. Rep..

[16] Blanco-Ulate B., Rolshausen P., Cantu D. (2013). Draft Genome Sequence of Neofusicoccum parvum Isolate UCR-NP2, a Fungal Vascular Pathogen Associated with Grapevine Cankers. Genome Announc..

[17] Abou-Mansour E., Débieux J.-L., Ramírez-Suero M., Bénard-Gellon M., Magnin-Robert M., Spagnolo A., Chong J., Farine S., Bertsch C., L’Haridon F. (2015). Phytotoxic metabolites from Neofusicoccum parvum, a pathogen of Botryosphaeria dieback of grapevine. Phytochemistry.

[18] Trotel-Aziz P., Mansour E.A., Courteaux B., Rabenoelina F., Clément C., Fontaine F., Aziz A. (2019). Bacillus subtilis PTA-271 Counteracts Botryosphaeria Dieback in Grapevine, Triggering Immune Responses and Detoxification of Fungal Phytotoxins. Front. Plant Sci..

[19] Pour F.N., Cobos R., Coque J.J.R., Serôdio J., Alves A., Félix C., Ferreira V., Esteves A.C., Duarte A.S. (2020). Toxicity of Recombinant Necrosis and Ethylene-Inducing Proteins (NLPs) from Neofusicoccum parvum. Toxins.

[20] Mehl J.W., Slippers B., Roux J., Wingfield M.J. (2017). Overlap of latent pathogens in the Botryosphaeriaceae on a native and agricultural host. Fungal Biol..

[21] Adams J., Lingbeck J., Crandall P., Martin E., O’Bryan C. (2015). Sweetgum: A new look. iForest Biogeosci. For..

[22] Gao L., Li Y., Xu Y., Hulcr J., Cognato A.I., Wang J.-G., Ju R.-T. (2017). Acanthotomicus sp. (Coleoptera: Curculionidae: Scolytinae), a New Destructive Insect Pest of North American Sweetgum Liquidambar styraciflua in China. J. Econ. Entomol..

[23] Kline K.L., Coleman M.D. (2010). Woody energy crops in the southeastern United States: Two centuries of practitioner experience. Biomass Bioenergy.

[24] Pedraza R., Williams-Linera G. (2003). Evaluation of native tree species for the rehabilitation of deforested areas in a Mexican cloud forest. New For..

[25] Lu S., Edwards M.C. (2016). Genome-Wide Analysis of Small Secreted Cysteine-Rich Proteins Identifies Candidate Effector Proteins Potentially Involved in Fusarium graminearum−Wheat Interactions. Phytopathology.

[26] Chen X.-R., Li Y.-P., Li Q.-Y., Xing Y.-P., Liu B.-B., Tong Y.-H., Xu J.-Y., And Y.T. (2015). SCR96, a small cysteine-rich secretory protein of P hytophthora cactorum, can trigger cell death in the Solanaceae and is important for pathogenicity and oxidative stress tolerance. Mol. Plant Pathol..

[27] Lyu X., Shen C., Fu Y., Xie J., Jiang D., Li G., Cheng J. (2016). A Small Secreted Virulence-Related Protein is Essential for the Necrotrophic Interactions of Sclerotinia sclerotiorum with Its Host Plants. PLoS Pathog..

[28] Wang D., Tian L., Zhang D., Song J., Song S., Yin C., Zhou L., Liu Y., Wang B., Kong Z. (2020). Functional analyses of small secreted cysteine-rich proteins identified candidate effectors in Verticillium dahliae. Mol. Plant Pathol..

[29] Odintsova T.I., Slezina M.P., Istomina E.A. (2020). Defensins of Grasses: A Systematic Review. Biomolecules.

[30] Slazak B., Kapusta M., Strömstedt A., Słomka A., Krychowiak-Masnicka M., Shariatgorji R., Andrén P.E., Bohdanowicz J., Kuta E., Göransson U. (2018). How Does the Sweet Violet (Viola odorata L.) Fight Pathogens and Pests—Cyclotides as a Comprehensive Plant Host Defense System. Front. Plant Sci..

[31] Su T., Han M., Cao D., Xu M. (2020). Molecular and Biological Properties of Snakins: The Foremost Cysteine-Rich Plant Host Defense Peptides. J. Fungi.

[32] Wong K.H., Tan W.L., Serra A., Xiao T., Sze S.K., Yang D., Tam J.P. (2016). Ginkgotides: Proline-Rich Hevein-Like Peptides from Gymnosperm Ginkgo biloba. Front. Plant Sci..

[33] Tapia-Tussell R., Lappe P., Ulloa M., Quijano-Ramayo A., Cáceres-Farfán M., Larqué-Saavedra A., Perez-Brito D. (2006). A rapid and simple method for DNA extraction from yeasts and fungi isolated from Agave fourcroydes. Mol. Biotechnol..

[34] Glass N.L., Donaldson G.C. (1995). Development of primer sets designed for use with the PCR to amplify conserved genes from filamentous ascomycetes. Appl. Environ. Microbiol..

[35] Sakalidis M., Ray J.D., Lanoiselet V., Hardy G., Burgess T.I. (2011). Pathogenic Botryosphaeriaceae associated with Mangifera indica in the Kimberley Region of Western Australia. Eur. J. Plant Pathol..

[36] Slippers B., Crous P.W., Denman S., Coutinho T.A., Wingfield B.D., Wingfield M.J. (2004). Combined Multiple Gene Genealogies and Phenotypic Characters Differentiate Several Species Previously Identified as Botryosphaeria dothidea. Mycologia.

[37] Daudi A., O’Brien J.A. (2012). Detection of Hydrogen Peroxide by DAB Staining in Arabidopsis Leaves. Bio-Protoc..

[38] Bozzola J.J., Russell L.D. (1999). Electron Microscopy: Principles and Techniques for Biologists.

[39] Emanuelsson O., Nielsen H., Brunak S., von Heijne G. (2000). Predicting Subcellular Localization of Proteins Based on their N-terminal Amino Acid Sequence. J. Mol. Biol..

[40] Omasits U., Ahrens C., Müller S., Wollscheid B. (2014). Protter: Interactive protein feature visualization and integration with experimental proteomic data. Bioinformatics.

[41] Armenteros J.J.A., Tsirigos K.D., Sønderby C.K., Petersen T.N., Winther O., Brunak S., Von Heijne G., Nielsen H. (2019). SignalP 5.0 improves signal peptide predictions using deep neural networks. Nat. Biotechnol..

[42] Armenteros J.J.A., Sønderby C.K., Sønderby S.K., Nielsen H., Winther O. (2017). DeepLoc: Prediction of protein subcellular localization using deep learning. Bioinformatics.

[43] Kulmanov M., Hoehndorf R. (2019). DeepGOPlus: Improved protein function prediction from sequence. Bioinformatics.

[44] Gasteiger E., Hoogland C., Gattiker A., Duvaud S., Wilkins M.R., Appel R.D., Bairoch A., Walker J.M. (2005). Protein Identification and Analysis Tools on the ExPASy Server. The Proteomics Protocols Handbook.

[45] Kumar S., Stecher G., Li M., Knyaz C., Tamura K. (2018). MEGA X: Molecular Evolutionary Genetics Analysis across Computing Platforms. Mol. Biol. Evol..

[46] Jones D.T., Taylor W.R., Thornton J.M. (1992). The rapid generation of mutation data matrices from protein sequences. Bioinformatics.

[47] Waterhouse A.M., Procter J.B., Martin D.M.A., Clamp M., Barton G.J. (2009). Jalview Version 2--a multiple sequence alignment editor and analysis workbench. Bioinformatics.

[48] Notredame C., Higgins D., Heringa J. (2000). T-coffee: A novel method for fast and accurate multiple sequence alignment. J. Mol. Biol..

[49] Thornton B., Basu C. (2011). Real-time PCR (qPCR) primer design using free online software. Biochem. Mol. Biol. Educ..

[50] Pfaffl M.W. (2001). A new mathematical model for relative quantification in real-time RT-PCR. Nucleic Acids Res..

[51] Vandesompele J.J., De Preter K., Pattyn F., Poppe B., Van Roy N., De Paepe A., Speleman F. (2002). Accurate normalization of real-time quantitative RT-PCR data by geometric averaging of multiple internal control genes. Genome Biol..

[52] Albuquerque G.M.R., Fonseca F.C.A., Boiteux L.S., Borges R.C.F., Miller R.N.G., Lopes C.A., Souza E.B., Fonseca M.E.N. (2021). Stability analysis of reference genes for RT-qPCR assays involving compatible and incompatible Ralstonia solanacearum-tomato ‘Hawaii 7996’ interactions. Sci. Rep..

[53] Czechowski T., Stitt M., Altmann T., Udvardi M., Scheible W.-R. (2005). Genome-Wide Identification and Testing of Superior Reference Genes for Transcript Normalization in Arabidopsis. Plant Physiol..

[54] Freeman S., Sharon M., Maymon M., Mendel Z., Protasov A., Aoki T., Eskalen A., O’Donnell K. (2013). Fusarium euwallaceae sp. nov.—A symbiotic fungus of Euwallacea sp., an invasive ambrosia beetle in Israel and California. Mycologia.

[55] Na F., Carrillo J., Mayorquin J.S., Ndinga-Muniania C., Stajich J.E., Stouthamer R., Huang Y.-T., Lin Y.-T., Chen C.-Y., Eskalen A. (2018). Two Novel Fungal Symbionts Fusarium kuroshium sp. nov. and Graphium kuroshium sp. nov. of Kuroshio Shot Hole Borer (Euwallacea sp. nr. fornicatus) Cause Fusarium Dieback on Woody Host Species in California. Plant Dis..

[56] Zhang Z.-N., Wu Q.-Y., Zhang G.-Z., Zhu Y.-Y., Murphy R.W., Liu Z., Zou C.-G. (2015). Systematic analyses reveal uniqueness and origin of the CFEM domain in fungi. Sci. Rep..

[57] Cram M., Coyle D., Spaine P., Lumpkin S., Coleman M. (2018). Fertilization and Irrigation Effect on Botryosphaeriaceae Canker Development in Intensively Managed Sweetgum (Liquidambar Styraciflua). Tree Plant. Notes.

[58] Hepting G.H. (1971). Diseases of Forest and Shade Trees of the United States.

[59] Amponsah N., Jones E., Ridgway H., Jaspers M.V. (2012). Microscopy of some interactions between Botryosphaeriaceae species and grapevine tissues. Australas. Plant Pathol..

[60] Pedraza J.M.T., Aguilera A.M., Díaz C.N., Ortiz D.T., Ponce G.V., Monter Á.V., López J.H. (2012). Identification, Pathogenicity, and Histopathology of Lasiodiplodia Theobromae on Mamey Sapote Grafts in Guerrero, México. Agrociencia.

[61] Van Loon L., van Strien E. (1999). The families of pathogenesis-related proteins, their activities, and comparative analysis of PR-1 type proteins. Physiol. Mol. Plant Pathol..

[62] De Lucca A.J., Cleveland T.E., Wedge D.E. (2005). Plant-derived antifungal proteins and peptides. Can. J. Microbiol..

[63] Molina A., Segura A., García-Olmedo F. (1993). Lipid transfer proteins (nsLTPs) from barley and maize leaves are potent inhibitors of bacterial and fungal plant pathogens. FEBS Lett..

[64] Jia Z., Gou J., Sun Y., Yuan L., Tang Q., Yang X., Pei Y., Luo K. (2010). Enhanced resistance to fungal pathogens in transgenic Populus tomentosa Carr. by overexpression of an nsLTP-like antimicrobial protein gene from motherwort (Leonurus japonicus). Tree Physiol..

[65] Zhang S., Wang X. (2017). One new kind of phytohormonal signaling integrator: Up-and-coming GASA family genes. Plant Signal. Behav..

[66] Nahirñak V., Almasia N.I., Hopp H.E., Vazquez-Rovere C. (2012). Snakin/GASA proteins: Involvement in Hormone Crosstalk and Redox Homeostasis. Plant Signal. Behav..

[67] Bindschedler L.V., Whitelegge J.P., Millar D.J., Bolwell G.P. (2006). A two component chitin-binding protein from French bean—association of a proline-rich protein with a cysteine-rich polypeptide. FEBS Lett..

[68] Massonnet M., Morales-Cruz A., Figueroa-Balderas R., Lawrence D.P., Baumgartner K., Cantu D. (2018). Condition-dependent co-regulation of genomic clusters of virulence factors in the grapevine trunk pathogen Neofusicoccum parvum. Mol. Plant Pathol..

[69] Rep M. (2005). Small proteins of plant-pathogenic fungi secreted during host colonization. FEMS Microbiol. Lett..

[70] Kulkarni R.D., Kelkar H.S., Dean A.R. (2003). An eight-cysteine-containing CFEM domain unique to a group of fungal membrane proteins. Trends Biochem. Sci..

[71] Fass D. (2012). Disulfide Bonding in Protein Biophysics. Annu. Rev. Biophys..

[72] Fass D., Thorpe C. (2018). Chemistry and Enzymology of Disulfide Cross-Linking in Proteins. Chem. Rev..

[73] Zhu W., Wei W., Wu Y., Zhou Y., Peng F., Zhang S., Chen P., Xu X. (2017). BcCFEM1, a CFEM Domain-Containing Protein with Putative GPI-Anchored Site, Is Involved in Pathogenicity, Conidial Production, and Stress Tolerance in Botrytis cinerea. Front. Microbiol..

[74] De Zwaan T.M., Carroll A.M., Valent B., Sweigard J.A. (1999). Magnaporthe grisea Pth11p Is a Novel Plasma Membrane Protein That Mediates Appressorium Differentiation in Response to Inductive Substrate Cues. Plant Cell.

[75] Vaknin Y., Shadkchan Y., Levdansky E., Morozov M., Romano J., Osherov N. (2014). The three Aspergillus fumigatus CFEM-domain GPI-anchored proteins (CfmA-C) affect cell-wall stability but do not play a role in fungal virulence. Fungal Genet. Biol..

