# Supplementary material for: Insights of the Neofusicoccum parvum–Liquidambar styraciflua Interaction and Identification of New Cysteine-Rich Proteins in Both Species
Source: J Fungi (Basel). 2021 Nov 30;7(12):1027. doi: 10.3390/jof7121027 (PMC8707630; doi:10.3390/jof7121027)
Supplement: Supplementary file 1 [file jof-07-01027-s001.zip › Figure S1.pdf]

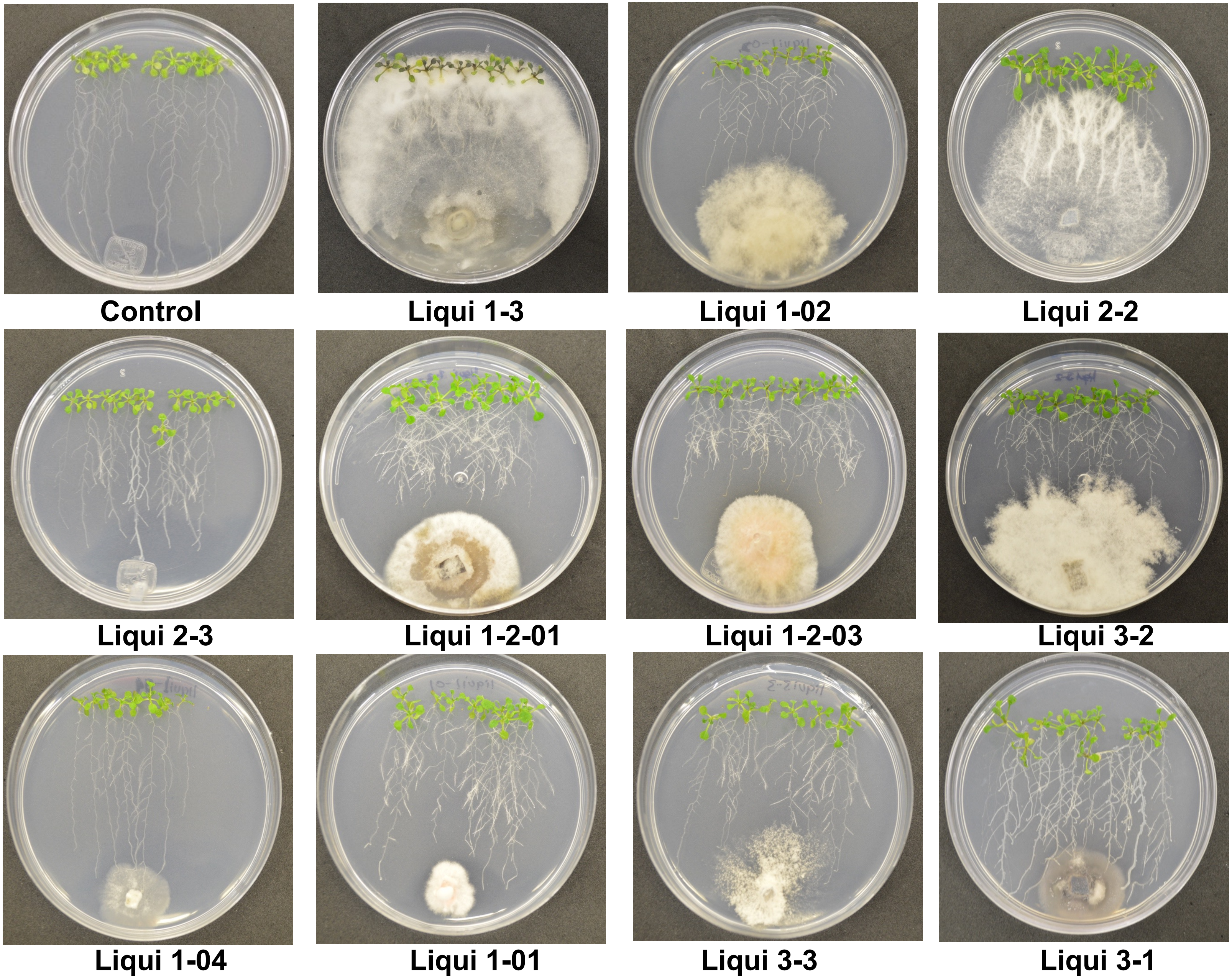

Figure S1. Pathogenicity screening in *A. thaliana* seedlings. 14-day-old *A. thaliana* seedlings infected with different fungal isolates. The photograph was taken at 7 dpi, and the data are representative of at least three independent experiments.
