# Supplementary material for: Insights of the Neofusicoccum parvum–Liquidambar styraciflua Interaction and Identification of New Cysteine-Rich Proteins in Both Species
Source: J Fungi (Basel). 2021 Nov 30;7(12):1027. doi: 10.3390/jof7121027 (PMC8707630; doi:10.3390/jof7121027)
Supplement: Supplementary file 1 [file jof-07-01027-s001.zip › Figure S2.pdf]

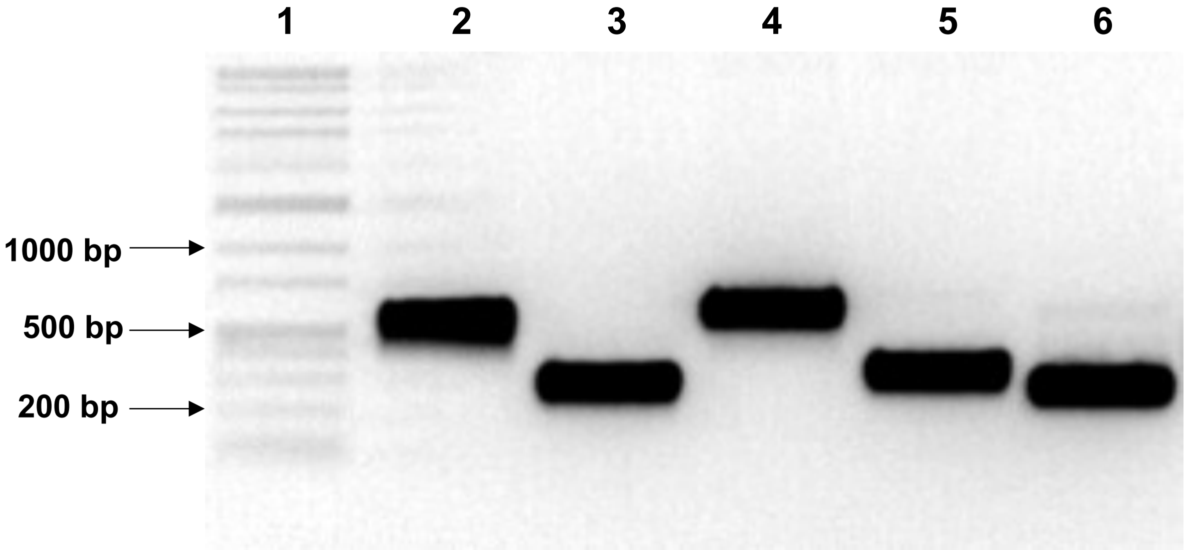

Figure S2. Molecular identification of the Liqui 1-3 strain. Agarose gel electrophoresis and PCR product bands. Lane 1: ladder, the primers used are listed; lane 2: Bt2a and Bt2b; lane 3: Np304-F and Np304-R; lane 4: RPB2bot7F and RPB2bot7F; lane 5: Bot15 and Bot16 and lane 6: EF1-728F and EF-BR.
