# Supplementary material for: Insights of the Neofusicoccum parvum–Liquidambar styraciflua Interaction and Identification of New Cysteine-Rich Proteins in Both Species
Source: J Fungi (Basel). 2021 Nov 30;7(12):1027. doi: 10.3390/jof7121027 (PMC8707630; doi:10.3390/jof7121027)
Supplement: Supplementary file 1 [file jof-07-01027-s001.zip › Figure S3.pdf]

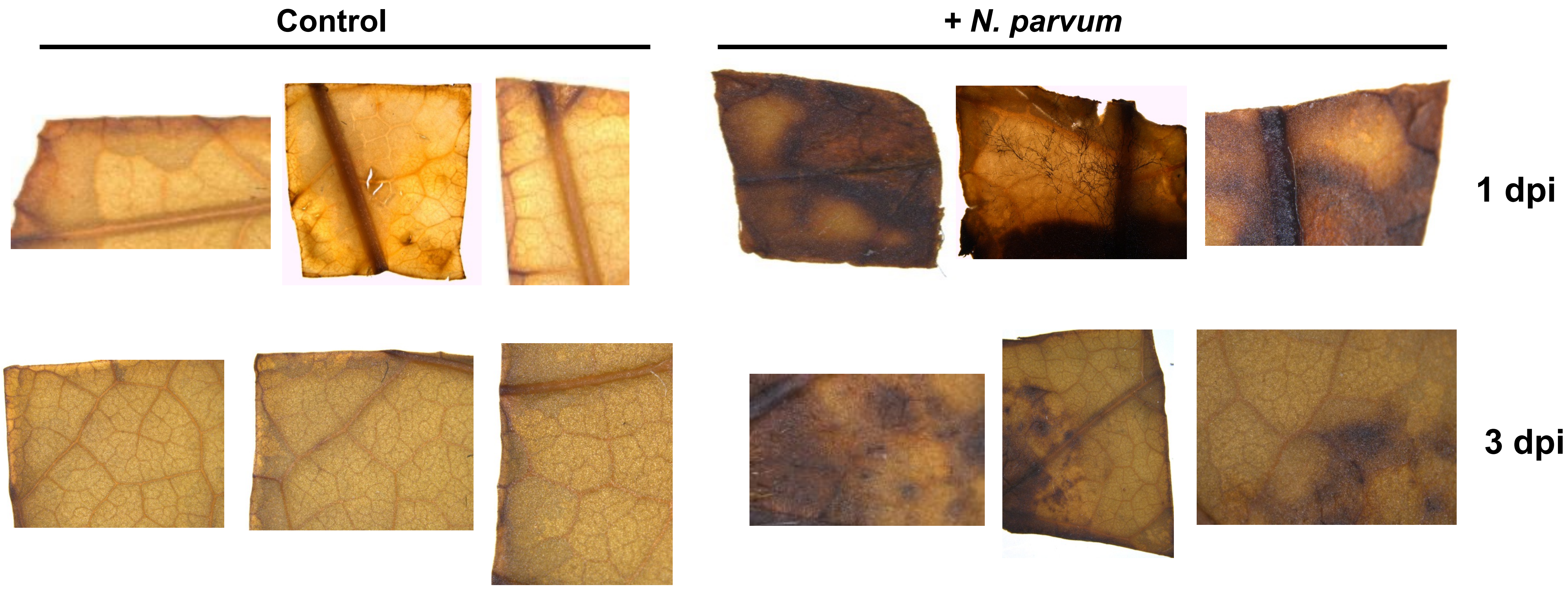

Figure S3. Detection of H_2O_2 by DAB staining of *L. styraciflua* leaves infected with *N. parvum* at early stages of infection. A stereomicroscope was used to acquire the images.
