# Supplementary material for: Insights of the Neofusicoccum parvum–Liquidambar styraciflua Interaction and Identification of New Cysteine-Rich Proteins in Both Species
Source: J Fungi (Basel). 2021 Nov 30;7(12):1027. doi: 10.3390/jof7121027 (PMC8707630; doi:10.3390/jof7121027)
Supplement: Supplementary file 1 [file jof-07-01027-s001.zip › Figure S4.pdf]

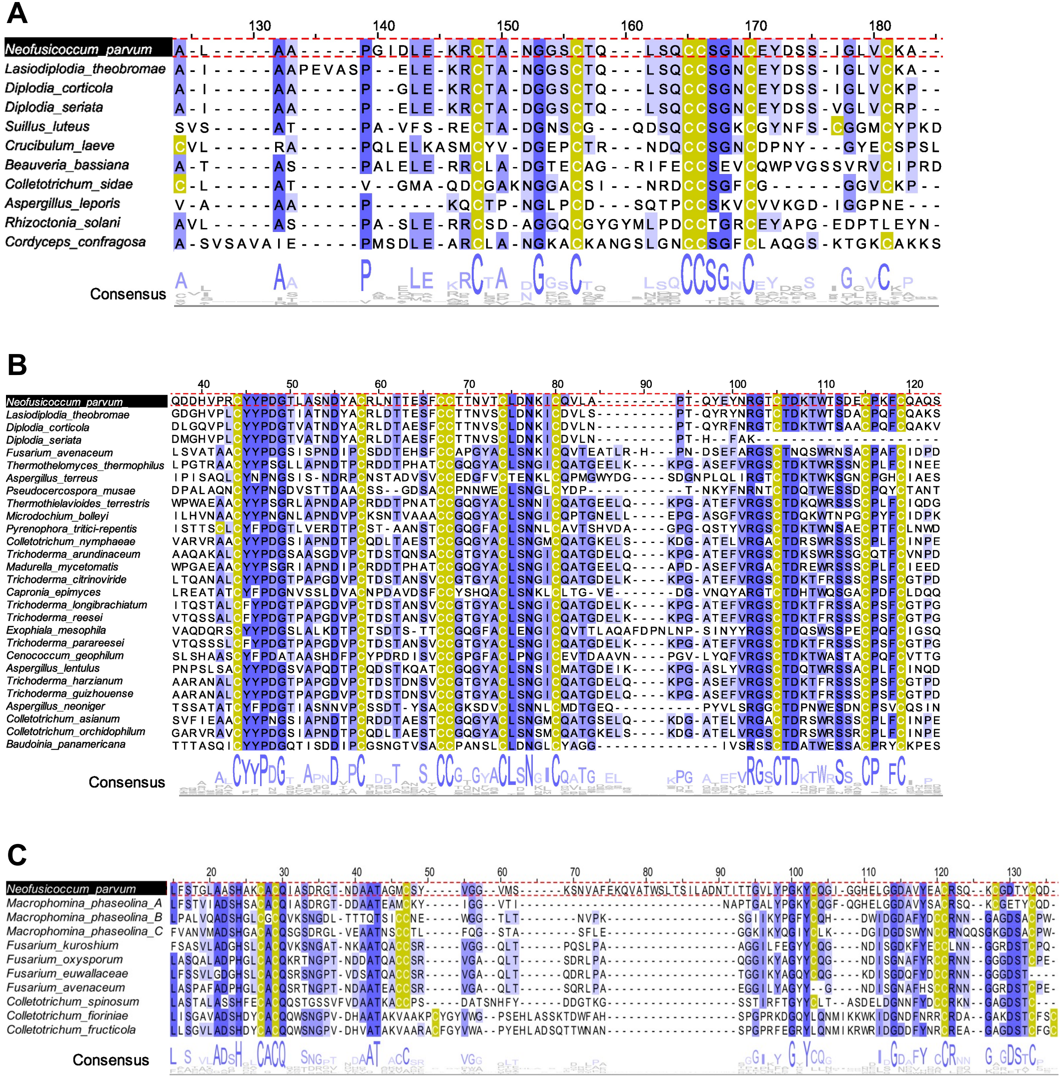

Figure S4. Multiple-sequence alignment of NpCysRPs and other orthologous sequences. A. NpCysRP1, **B.** NpCysRP2 and **C.** NpCysRP3. Partial protein sequences were aligned using Jalview software and the Tcoffee method. The conserved cysteine residues are highlighted in yellow. The blue shading intensity reflects the level of amino acid identity at each position.

The access numbers of each sequence are: **A.** *N. parvum* (EOD50423.1), *L. theobromae* (KAB2569989.1), *D. corticola* (XP_020126718.1), *D. seriata* (KKY27788.1), *S. luteus* (KIK46160.1), *C. leave* (TFK32468.1), *B. bassiana* (PQK10211.1), *C. sidae* (TEA07484.1), *A. leporis* (KAB8069877.1), *R. solani* (CUA75287.1), *C. confragosa* (OAA70963.1). **B.** *N. parvum* (EOD50922.1), *L. theobromae* (KAB2578904.1), *D. corticola* (XP_020125849.1), *D. seriata* (KKY21900.1), *F. avenaceum* (KIL85060.1), *T. thermophilusz* (XP_003666291.1), *A. terreus* (XP_001217394.1), *P. musae* (KXT13283.1) *T. terrestris* (XP_003656631.1), *P. tritici-repentis* (XP_001934627.1), *C. nymphaeae* (KXH38321.1), *M. mycetomatis* (KXX77126.1), *T. citrinoviride* (XP_024746670.1), *C. epimyces* (XP_007733601.1), *T. longibrachiatum* (PTB72285.1), *T. reesei* (XP_006969324.1), *E. mesophila* (RVX68508.1), *T. parareesei* (A9Z42_0055550), *A. lentulus* (KKO98531.1), *T. harzianum* (XP_024771023.1), *T. guizhouense* (PB44849.1), *A. neoniger* (XP_025474954.1), *C. asianum* (KAF0319766.1), *C. orchidophilum* (XP_022481280.1), *B. panamericana* (XP_007680904.1). **C.** *N. parvum* (EOD52838.1), *M. phaseolina* A (EKG20686.1), *M. phaseolina* B (EKG22346.1), *M. phaseolina* C (EKG22347.1), *F. euwallaceae* (RTE68176.1), *F. kuroshium* (RMI89680.1), *Fusarium* sp. AF-3 (RSL38373.1), *F. avenaceum* (KIL83641.1), *F. oxysporum* (EXK86396.1), *C. fioriniae* (EXF77634.1), *C. fructicola* (XP_031888108), *C. spinosum* (TDZ33147).
