# Supplementary material for: Insights of the Neofusicoccum parvum–Liquidambar styraciflua Interaction and Identification of New Cysteine-Rich Proteins in Both Species
Source: J Fungi (Basel). 2021 Nov 30;7(12):1027. doi: 10.3390/jof7121027 (PMC8707630; doi:10.3390/jof7121027)
Supplement: Supplementary file 1 [file jof-07-01027-s001.zip › Figure S6.pdf]

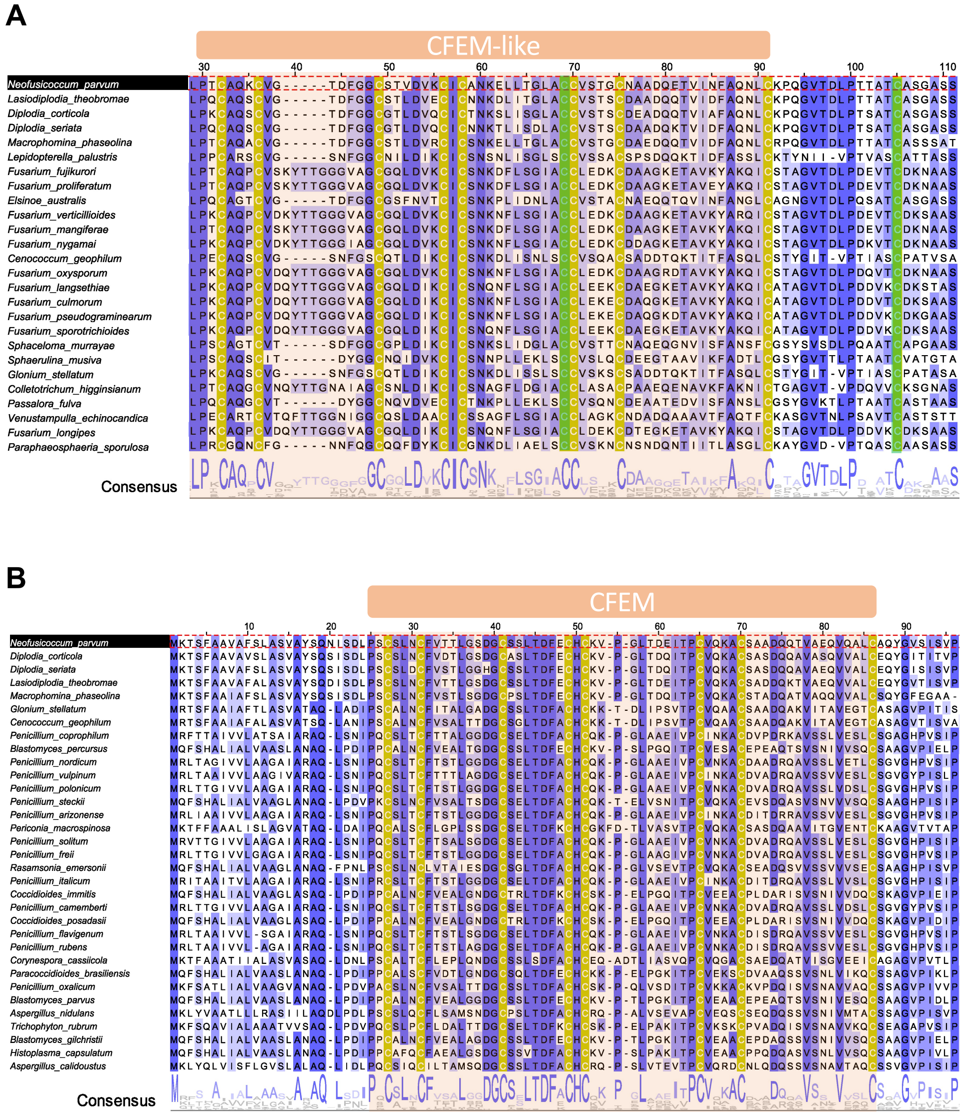

Figure S6. Multiple-sequence alignment of NpCysRPs and other orthologous sequences. **A.** NpCysRP4, **B.** NpCysRP5. Partial protein sequences were aligned using Jalview software and the Tcoffee method. The conserved cysteine residues are highlighted in yellow, and the cysteines that are not canonical to the CFEM domain are highlighted in green. The blue shading intensity reflects the level of amino acid identity at each position. The CFEM and CFEM-like domains are highlighted in orange. The access numbers of each sequence are: **A.** *N. parvum* (EOD51105.1), *L. theobromae* (KAB2571564.1), *D. corticola* (XP_020129061.1), *D. seriata* (KKY13873.1), *M. phaseolina* (EKG14570.1), *L. palustris* (OCK84136.1), *F. fujikurori* (QGI58805.1), *F. proliferatum* (XP_031075444.1), *E. australis* (TKX25331.1), *F. verticillioides* (RBR06989.1), *F. mangiferae* (CVK83605.1), *F. nygamai* (PNP52701.1), *C. geophilum* (OCK98732.1), *F. oxysporum* (EXA53032.1), *F. langsethiae* (KPA46759.1), *F. culmorum* (PTD11477.1), *F. pseudograminearum* (XP_009256009.1), *F. sporotrichioides* (RGP70600.1), *S. murrayae* (PNS17704.1), *S. musiva* (XP_016758053.1), *G. stellatum* (OCL13334.1), *C. higginsianum* (TIC93879.1), *P. fulva* (AQA29231.1), *V. echinocandica* (RDL36609.1), *F. longipes* (RGP60032.1), *P. sporulosa* (XP_018029092.1). **B.** *N. parvum* (EOD44996.1), *D.corticola* (XP_020133845.1), *D. seriata* (KKY24076.1), *L. theobromae* (KAB2577423.1), *M. phaseolina* (EKG19367.1), *G. stellatum* (OCL07361.1), *C. geophilum* (OCK97775.1), *P. coprophilum* (OQE36870.1), B. percursus (OJD22636.1), *P. nordicum* (KOS37798.1), *P. vulpinum* (OQE12392.1), *P. polonicum* (OQD69393.1), *H. capsulatum* (EEH05575.1), *P. steckii* (OQE27319.1), *P. arizonense* (XP_022485186.1), *P. macrospinosa* (PVI08476.1), *P. solitum* (OQD85155.1), *P. freii* (KUM58798.1), *R. emersonii* (XP_013330685.1), *P. italicum* (KGO74509.1), *C. immitis* (XP_001240075.1), *P. camemberti* (CRL28928.1), *C. posadasii* (XP_003069153.1), *P. flavigenum* (OQE32686.1) , *P. rubens* (XP_002567673.1), *C. cassiicola* (PSN73263.1), *P. brasiliensis* (ODH22777.1) , *P. oxalicum* (EPS32950.1), *B. parvus* (PGH03478.1), *A. nidulans* (XP_660049.1), *T. rubrum* (XP_003231199.1), *B. gilchristii* (XP_002621720.1), *H. capsulatum* (XP_001539462.1), *A. calidoustus* (CEL09455.1).
