# Supplementary material for: Insights of the Neofusicoccum parvum–Liquidambar styraciflua Interaction and Identification of New Cysteine-Rich Proteins in Both Species
Source: J Fungi (Basel). 2021 Nov 30;7(12):1027. doi: 10.3390/jof7121027 (PMC8707630; doi:10.3390/jof7121027)
Supplement: Supplementary file 1 [file jof-07-01027-s001.zip › Table S1.pdf]

Table S1. Primers used in this study for the molecular identification of Liqui 1-3 strain and qRT-PCR experiments.

Target gene	Primers	Primer sequence (5`- 3`)	Size (bp)	References
b-tubulin	Bt2a	GGT AACCAAATCGGTGCTGCTTTC	450-500	Glass and Donaldson, 1995
	Bt2b	ACCCTCAGTGTAGTGACCCTTGGC		
ITS	Np304-F	TGAACTTCGCAGTCTGAA	304	Cheng-nang X et al. 2016
	Np304-R	CTCCAAAGCGAGGTGTTT		
RPB2	RPB2bot6F	GGTAGCGACGTCACTCCC	500	Sakalidis et al. 2011
	RPB2bot7F	GGATGGATCTCGCAATGCG		
Unknown locus containing microsatellite	Bot15	CTGACTTGTGACGCCGGCTC	350	Slippers et al. 2004
	Bot16	CAACCTGCTCAGCAAGCGAC		
EF-1a	EF1-728F	CATCGAGAAGTTCGAGAAGG	300	Carbone et al. 1999
	EF-BR	CRA TGG GTG ATA CCA RCG CTC		Sakalidis et al. 2011
NpCysP1	For	GGCTTCCAAGAAGGAGAAGAG	108	
	Rev	CAAACAAGGCCAATGGAAGAG		
NpCysP2	For	CAACACTACCGAGTCCTTCTG	121	
	Rev	TCGGACGTCCAGGTCTTAT		
NpCysP3	For	CGGCGGTGTTATGAGTAAGT	84	
	Rev	GTGTTGTCCGCTAGGATAGAAG		
NpCysP4	For	ATCTGCGCCAACAAGGAA	98	
	Rev	TTCTGCGCGAAGTTGATGA		
NpCysP5	For	TCTCCCTGGCCTCTGTT	75	
	Rev	TGGTGACGAAGCAGTTGAG		
LsCysP1	For	GCAAACCTCTGGAATGATGGA	118	
	Rev	AAACAAACGGAGATACGGTCA		
LsCysP2	For	TTATGCTGCCGCTGAACTTG	99	
	Rev	CCTTTGCACGAGACTGCCTA		
LsCysP3	For	CACCACACAAGCAAGGTTAAG	121	
	Rev	CCCTTTCCACTCACAGATCAA		
LsCysP4	For	ACAAGCAAGGGACAGGAAGG	101	
	Rev	TTGGAAGGACTGGGAAGGAA		
LsCysP5	For	CTTGCTTCACTCCTCGTTTCT	104	
	Rev	GAACTCTTGGGAGGAGAACTTG		
