# Supplementary material for: Insights of the Neofusicoccum parvum–Liquidambar styraciflua Interaction and Identification of New Cysteine-Rich Proteins in Both Species
Source: J Fungi (Basel). 2021 Nov 30;7(12):1027. doi: 10.3390/jof7121027 (PMC8707630; doi:10.3390/jof7121027)
Supplement: Supplementary file 1 [file jof-07-01027-s001.zip › Table S2.pdf]

Table S2. CysRP nucleotide sequences.

Assigned name	Target name	Length cDNA	Coding sequence	Length coding sequence
LsCysRP1	Liquidambar_styraciflua_01052015_comp30850_c0_seq1	1326	ATGGACCGATTGAGCAGCATGCGCTCTGTATTTTGGATTTTGGTCTCCTTTGGAACAA<br>ACGCAGCTGTCAGTGAGTTGATAAATGTTGCCTGCCCCCAGAGTGCAGAAGTGGACC<br>ATTGCATGTGTAATTGTAGGGCAATAGACGATGCTTGGAATGTTGATTGGCATAATGA<br>CCTGCGGATGTTTGGCTTGAAAACTGAAATACAAAGGCCTCAAAATGAAGTGTGTTG<br>CAATTTGGTAACCAGTTGTGTATTTATACATTGGGATCTTGTTTCTCACAGGAAAAGTC<br>GGAACTTAAGATGA	303
LsCysRP2	Liquidambar_styraciflua_01052015_comp28855_c0_seq1	1868	ATGGGCATGGCATTGTTCTGCCCAGTAAGTGGGTTGCAGCAGCAGATAACTGCTGCA<br>ACCTCAAAGTTTGATGGCAAGGGAACAAGTGGGGTTGGTGCTTGTTGCACCAAACCC<br>TGCCCTTGCCAGCACCAAATGAAGAAGCTCTGTTGCATTAGCTCTTATGGTTACTGCA<br>ATAGGTTTCGGTTTAAGGAATTGTCCTACCACCATTTGGTTGCTAAGGCAAATCATTCT<br>TGGCTCAGCTGCATTGAGGTCATCTTTGCCATTCCATACAGCTCCGCTGACCTGGGGT<br>GGTTAGAGATGGTCAAACCTTAA	312
LsCysRP3	Liquidambar_styraciflua_01052015_comp18453_c1_seq1	560	ATGGCTAGCTCAGGAATCCTTAACCTTGCTTGTGTGGTGCTCGTCAGCATGGTGTTGGT<br>TGCACCCCATGCTGAAGCTGCCATGACATGTGGTCAGGTGACGAGTAGCGTGTCCCC<br>ATGCCTCCCCTACCTGAAGGGTGCTAGCCCATTGCAGCCAGGCTGCTGCAATGGGATC<br>AGGTCCCTCAACAGCGCGGCTAAGACCACACCGGACCGCCAGGCTGCTTGTAGGTGC<br>TTGCAGCAGGCTGCTTCTAGCATCTCTGGTATCAATTTGGGTCTTGCATCTGGCCTTCC<br>TGGCAAATGTGGTATCAGCATT	312
LsCysRP4	Liquidambar_styraciflua_01052015_comp37785_c0_seq1	1655	ATGGGTTTTACAGGAATGTCTCCAATGAGCTTCCTATGTGCTTGGTGCTGCGGTCAAC<br>CAACTCATCATCTTGAAGCATTAGTCAGTTGGACAGCAGCGTTGCTTCAATCATTAAA<br>GCTCAAAGTTGCTGAGGACACAGATTTTTTTACTAATAGTTTTGATCCTGTAGAAGTAT<br>GGATCATTTCTTTGTACAAATATTTGTATCATAAGACATTAAGAAAGACCAACATCGG<br>CAAAGAAGCTGCACTGAAGAGGGCTAAGCCTATGATGATTGCTTGTGTTGTAGAGAT<br>GCTGCTGATAGATTTGCAGTCTTGA	315
LsCysRP5	Liquidambar_styraciflua_01052015_comp31840_c0_seq3	765	ATGGCCATCTACAAGGCTCTTCTTGCTTCACTCCTCGTTTCTCTTTTTGTTCTCCATCTTG<br>TTGTAGCTGATGGAATGGGGATGCAGACAACGGATGCTGCAACAAGTTCTCCTCCCA<br>AGAGTTCTCCCCCCAAGAAAATTGATTGTGGAGGAGCTTGCTCCTCTAGGTGCCAGTT<br>ATCGTCGAGGCCAAACCTATGCAAGAGGGCGTGTGGGACTTGCTGTGCCCGTTGCAA<br>CTGCGTTCCTCCGGGTACTTCCGGGAATGAAGATGTCTGTCCCTGCTACGCCAGCATG<br>ACCACCCACGGCGGAAGGCACAAGTGCCCTTGA	324
NpCysRP1	Neofusicoccum parvum UCRNP2 chromosome Unknown NP2_03_scaffold_347, whole genome shotgun sequence	KB915985.1	ATGCAGTTCAAGTTCGCCCTTGTCTCCCTCTTCACCGCCCTGGCCCTTGCCGCGCCCGG<br>CATCGATCTCGAGAAGAGGTGCACCGCTAACGGCGGCTCTTGCACCCAGCTGAGCCA<br>GTGCTGCAGTGGCAACTGCGAGTACGACTCTTCCATCGGTCTTGTGTGCAAGGCGGCT<br>TCCAAGAAGGAGAAGAGGTGCACGGCCAACGGCGGCGCCTGCACTCAGTTGGCTCA<br>GTGCTGCAGTGGTAACTGCGAGTACGACTCTTCCATTGGCCTTGTTTGCAAGCCCTAG	288
NpCysRP2	Neofusicoccum parvum UCRNP2 chromosome Unknown NP2_03_scaffold_285, whole genome shotgun sequence	KB915923.1	ATGCGACCACTGTACAGCCACCTTGTACACACCCTACCTCCTCTCTTCTCCATCCTCCT<br>CGACCCCCGCCCCGCGTCTGCACAGGACGACCACGTCCCCCGCTGCTACTACCCCGAC<br>GGCACGCTCGCGTCCAACGACTACGCCTGCCGGCTCAACACTACCGAGTCCTTCTGCT<br>GCACGACCAACGTCACATGCCTCGACAACAAGATCTGCCAGGTGCTGGCGCCCACGC<br>AGTACGAGTACAACCGCGGCACCTGCACCGATAAGACCTGGACGTCCGACGAGTGC<br>CCCAAGTTCTGTCAAGCGCAATCGCCGTCGTACGGCTCGGGGGTGATCAGACCTGTAT<br>ACTGA	351
NpCysRP3	Neofusicoccum parvum UCRNP2 chromosome Unknown NP2_03_scaffold_45, whole genome shotgun sequence	KB915683.1	ATGCGTTTCTTCGTTGCTATCTCCGTCGCCGCCACGGCCCTCTTCTCTACCGGCCTTGCC<br>GCCTCGCACGCTAAGTGTGCCTGCCAGATTGCCTCTGACCGCGGAACCAACGATGCC<br>GCTACTGCTGGCATGTGCTCCTACGTCGGCGGTGTTATGAGTAAGTCCAACGTGGCGT<br>TCGAAAAGCAAGTTGCCACGTGGTCGCTGACTTCTATCCTAGCGGACAACACCATCA<br>CTACGGGCGTTCTTTACCCCGGCAAATACTGCCAGGGCATTGGCGGCCACGAGCTCG<br>GCGGTGACGCTGTCTACGAGGCTTGCCGTAGCCAGAAGTGCGGTGATACCTACTGCC<br>AGGACTCCACTTGCGTCTAA	366
NpCysRP4	Neofusicoccum parvum UCRNP2 chromosome Unknown NP2_03_scaffold_259, whole genome shotgun sequence	KB915897.1	ATGCGCGTCTCCGCATTTGCCGCCATCCTCGGCTATAGCAGCCTCGCTCTTGCTCAGAC<br>ACAGCTGCTCCCTACCTGCGCTCAAAAATGCGTTGGCACCGATTTCGGCGGCTGCTCC<br>ACGGTTGATGTCAAGTGCATCTGCGCCAACAAGGAACTGCTCACGGGCCTTGCCTGCT<br>GCGTGTCGACCGGCTGCAATGCCGCGGACCAGGAGACCGTCATCAACTTCGCGCAGA<br>ACCTGTGCAAGCCGCAGGGCGTGACCGACCTCCCCACGACGGCCACCTGCGCGTCGG<br>GCGCCTCTTCGGCCACCAGCTCTGCCTCCTCCGGCACCGCGTCCACCACTGCGTCCAG<br>CGCCGCCAGCGCTTCCGCTTCGGCGACCGGCTCCGCTGCCAGCTCTGCCTCCTCCGCT<br>GCGTCGTCGGTTGCGTCCAGCGCCGCGTCGGCCGCGAGCAGTGCCGCGGCTTCTGCCT<br>CTGCTGCCACCACTGCCGGCAGCGGCGCGGGTTCGTGCCAGGGCAACGCGGCGGGTA<br>TGGGATTCGGTCTTGTGCTTGCTGGTCTGCTGGGCGCGCTCTAG	564
NpCysRP5	Neofusicoccum parvum UCRNP2 chromosome Unknown NP2_03_scaffold_1007, whole genome shotgun sequence	KB916645.1	ATGAAGACCTCCTTCGCCGCCGTTGCCTTCTCCCTGGCCTCTGTTGCCTACTCCCAGAA<br>CATCTCGGACCTCCCCAGCTGCTCGCTCAACTGCTTCGTCACCACCCTCGGCAGTGAC<br>GGCTGCTCGTCGCTCACCGACTTCGAGTGCCACTGCAAGGTCCCCGGCCTGACCGACG<br>AGATCACTCCCTGCGTCCAGAAGGCTTGCTCCGCCGCCGACCAGCAGACCGTTGCCG<br>AGCAGGTCCAGGCCCTGTGCGCCCAGTACGGCGTCAGCATCTCGGTCCCTGAGGCGT<br>CCTCGACGGCAGCTCCGTCCTCGACCGCCGCCGCCACCAGCGCTCCGGCCTCGTCCTC<br>TGCTGCGGCCACCACGGCGGCTTCGTCGGCTGCGTCCAGCGCTGCGTCCAGCGCCAG<br>CGCCTCTGCCACCAGCACCGAGACTGCCTCTCCCTCGGCCACTGACGTCGTCATCTCC<br>ACCCCGGTCGCCACCCCGACCACCCCCGGCGCTTCCACTCCCGCCGGCTCCACCCCGA<br>CTCCCTCTCAGTTCCAGGGTGCCGCTGGCAAGACTGGTGTCGTTGGCGGCGTTGTCGG<br>TTTCGCCGCCGCCGTCGCTGCTGTTGCCGCCCTCTAA	615
