# Supplementary material for: Insights of the Neofusicoccum parvum–Liquidambar styraciflua Interaction and Identification of New Cysteine-Rich Proteins in Both Species
Source: J Fungi (Basel). 2021 Nov 30;7(12):1027. doi: 10.3390/jof7121027 (PMC8707630; doi:10.3390/jof7121027)
Supplement: Supplementary file 1 [file jof-07-01027-s001.zip › Table S3.pdf]

Table S3. Predicted disulfide bonds for CysRPs.

Assigned name	Total cysteines	Cysteine position in the amino acid sequence	Cyscon[1]	DISULFIND[2]	DiANNA[3]	CYS_REC[4]	SCRATCH[5]
LsCysRP1	7	31, 40, 42, 44, 76, 77, 83	31-83<br>40-77<br>44-76	40 - 76<br>42 - 77<br>44 - 83	40 - 76<br>42 - 44<br>77 - 83	40-76<br>42-83<br>44-77	77 - 83<br>31 - 40<br>44 - 76
LsCysRP2	9	34, 35, 39, 41, 49, 50, 57, 81	34 - 41<br>35 - 50<br>49 - 57	34 - 41<br>35 - 39<br>49 - 81<br>50 - 57	34 - 57<br>35 - 49<br>39 - 41<br>50 - 81	34-39<br>41-49<br>50-60	35 - 49<br>31 - 57<br>34 - 50
LsCysRP3	8	30, 40, 54, 55, 75, 77, 100	30 - 77<br>40 - 54<br>75 - 100	40 - 55<br>54 - 75<br>77 - 100	30 - 77<br>40 - 54<br>55 - 100	30 - 54<br>40 - 77<br>55 - 100	54 - 75<br>55 - 100<br>11 - 30
LsCysRP4	4	13, 16, 17, 93	___	___	___	___	___
LsCysRP5	12	49, 53, 57, 66, 70, 73, 74, 77, 79, 91, 93, 106	49 - 77<br>53 - 70<br>57 - 66<br>73 - 93<br>74 - 106<br>79 - 91	Connectivity Pattern can be predicted for up to 5 bonds.	49 - 93<br>53 - 73<br>57 - 66<br>70 - 106<br>74 - 79<br>77 - 91	49 - 74<br>53 - 73<br>57 - 77<br>66 - 79<br>70 - 91	49 - 53<br>77 - 93<br>74 -106<br>79 - 91<br>57 - 70
NpCysRP1	12	27, 34, 40, 41, 45, 55, 65, 72, 78, 79, 83, 93	27 - 55<br>34 - 83<br>40 - 72<br>41 - 78<br>45 - 79<br>79 - 93	Connectivity Pattern can be predicted for up to 5 bonds.	Many posibel connectivity	27 - 40<br>34 - 78<br>41 - 79<br>45 - 55<br>65 - 83<br>72 - 93	34 - 45<br>78 - 93<br>27 - 41<br>40 - 55<br>72 - 83<br>65 - 79
NpCysRP2	9	35, 49, 58, 59, 65, 71, 86, 96, 100	35 - 49<br>59 - 86<br>65 - 71<br>96 - 100	49 - 86<br>58 - 96<br>59 - 65<br>71 - 100	35 - 59<br>58 - 71<br>65 - 100<br>86 - 96	49 - 71<br>58 - 100<br>59 - 86<br>65 - 96	49 - 65<br>86 - 96<br>58 - 71<br>35 - 59
NpCysRP3	8	26, 28, 45, 88, 105, 110, 115, 120	28 - 105<br>45 - 88<br>110 - 120	26 - 120<br>28 - 45<br>88 - 115<br>105 - 110	26 - 28<br>45 - 120<br>88 - 110<br>105 - 115	28 - 105<br>45 - 115<br>88 - 110	88 - 110<br>105 - 115<br>28 - 45
NpCysRP4	11	26,30,38,45,47,58,59,64, 80, 94, 166	26 - 45<br>30 - 57<br>38 - 58<br>59 - 80<br>94 - 166	26 - 30<br>38 - 166<br>45 -58<br>47 -64<br>59 -80	26 - 59<br>38 - 166<br>45 - 80<br>47 - 58<br>64 - 94	26 - 45<br>30 - 59<br>38 - 58<br>64 - 94<br>80 - 166	30 - 47<br>94 - 167<br>26 - 45<br>38 - 64<br>59 - 80
NpCysRP5	8	27, 31, 41, 49, 51, 63, 68, 84	27 - 63<br>41 - 51<br>68 - 84	27 - 63<br>31 - 68<br>41 - 84<br>49 - 51	27 - 49<br>31 - 68<br>41 - 63<br>51 - 84	27-63<br>31-41<br>51-68	51 - 63<br>68 - 84<br>41 - 49

[1]Jing Yang^, Bao-Ji He^, Richard Jang, Yang Zhang, and Hong-Bin Shen, Bioinformatics, 2015, 31: 3773-3781.

[2]A. Ceroni, A. Passerini, A. Vullo and P. Frasconi, Nucleic Acids Research, 2006, 34(Web Server issue):W177–W181.

[3]F. Ferre and P. Clote, Nucleic Acids Res. 2006 Jul 1; 34(Web Server issue): W182–W185.

[4]Softberry Web Site http://www.softberry.com/berry.phtml?topic=cys_rec&group=programs&subgroup=propt

[5]Scratch Proteína Prediction http://scratch.proteomics.ics.uci.edu/index.html
