# Supplementary figures and images for: Insights of the Neofusicoccum parvum–Liquidambar styraciflua Interaction and Identification of New Cysteine-Rich Proteins in Both Species
Source: J Fungi (Basel). 2021 Nov 30;7(12):1027. doi: 10.3390/jof7121027 (PMC8707630; doi:10.3390/jof7121027)

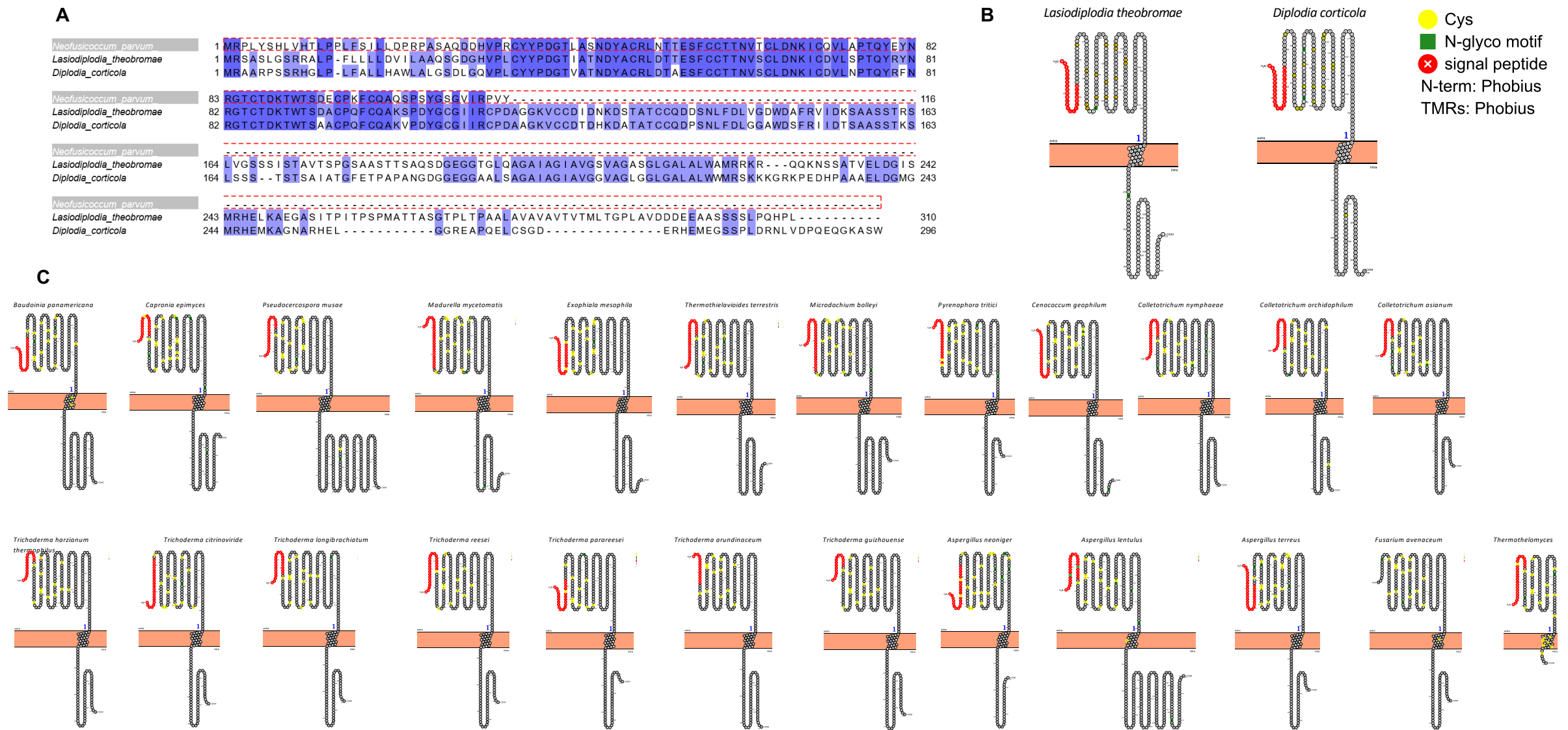

Supplement: Supplementary file 1 [file jof-07-01027-s001.zip › Figure S5.pdf]
